# Predicting recovery in patients with mild traumatic brain injury and a normal CT using serum biomarkers and diffusion tensor imaging (CENTER-TBI): an observational cohort study

**DOI:** 10.1016/j.eclinm.2024.102751

**Published:** 2024-08-08

**Authors:** Sophie Richter, Stefan Winzeck, Marta M. Correia, Endre Czeiter, Daniel Whitehouse, Evgenios N. Kornaropoulos, Guy B. Williams, Jan Verheyden, Tilak Das, Olli Tenovuo, Jussi P. Posti, Anne Vik, Kent Gøran Moen, Asta K. Håberg, Kevin Wang, Andras Buki, Andrew Maas, Ewout Steyerberg, David K. Menon, Virginia F.J. Newcombe, Collaborative European NeuroTrauma Effectiveness Research in Traumatic Brain Injury Magnetic Resonance Imaging (CENTER-TBI MRI) Substudy Participants and Investigators, Krisztina Amrein, Nada Andelic, Lasse Andreassen, Audny Anke, Philippe Azouvi, Bo-Michael Bellander, Habib Benali, Andras Buki, Alessio Caccioppola, Emiliana Calappi, Marco Carbonara, Giuseppe Citerio, Hans Clusmann, Mark Coburn, Jonathan Coles, Marta Correia, Endre Czeiter, Véronique De Keyser, Vincent Degos, Bart Depreitere, Live Eikenes, Erzsébet Ezer, Kelly Foks, Shirin Frisvold, Damien Galanaud, Alexandre Ghuysen, Ben Glocker, Asta Haberg, Iain Haitsma, Eirik Helseth, Peter J. Hutchinson, Evgenios Kornaropoulos, Noémi Kovács, Ana Kowark, Steven Laureys, Didier Ledoux, Hester Lingsma, Andrew I.R. Maas, Geoffrey Manley, David K. Menon, Tomas Menovsky, Benoit Misset, Visakh Muraleedharan, Ingeborg Nakken, Virginia Newcombe, Wibeke Nordhøy, József Nyirádi, Fabrizio Ortolano, Paul M. Parizel, Vincent Perlbarg, Paolo Persona, Wilco Peul, Jussi P. Posti, Louis Puybasset, Sophie Richter, Cecilie Roe, Olav Roise, Rolf Rossaint, Sandra Rossi, Daniel Rueckert, Ranjit D. Singh, Toril Skandsen, Abayomi Sorinola, Emmanuel Stamatakis, Ewout W. Steyerberg, Nino Stocchetti, Riikka Takala, Viktória Tamás, Olli Tenovuo, Zoltán Vámos, Gregory Van der Steen, Inge A. van Erp, Wim Van Hecke, Thijs Vande Vyvere, Jan Verheyden, Anne Vik, Victor Volovici, Lars T. Westlye, Daniel Whitehouse, Guy Williams, Stefan Winzeck, Peter Ylén, Tommaso Zoerle

**Affiliations:** a Department of Medicine, University of Cambridge, Cambridge, UK; b MRC Cognition and Brain Sciences Unit, University of Cambridge, Cambridge, UK; c Neurotrauma Research Group, Szentágothai Research Centre, University of Pécs, Pécs, Hungary; d Department of Neurosurgery, Medical School, University of Pécs, Pécs, Hungary; e HUN-REN-PTE Clinical Neuroscience MR Research Group, University of Pécs, Pécs, Hungary; f Wolfson Brain Imaging Centre, Department of Clinical Neurosciences, University of Cambridge, Cambridge, UK; g Research and Development, icometrix, Leuven, Belgium; h Department of Radiology, Addenbrooke's Hospital, Cambridge, UK; i Turku Brain Injury Center, Turku University Hospital & University of Turku, Turku, Finland; j Department of Neurourgery, Turku University Hospital & University of Turku, Turku, Finland; k Department of Neuromedicine and Movement Science, Norwegian University of Science and Technology (NTNU), Trondheim, Norway; l Department of Neurosurgery, St. Olavs Hospital, Trondheim University Hospital, Trondheim, Norway; m Department of Circulation and Medical Imaging, Norwegian University of Science and Technology, Trondheim, Norway; n Department of Radiology and Nuclear Medicine, St.Olavs Hospital, Trondheim University Hospital, N-7006, Trondheim, Norway; o Department of Radiology, Vestre Viken Hospital Trust, Drammen Hospital, Drammen, Norway; p Center for Medical Equipment, Technology and Innovation, St. Olavs Hospital, Trondheim University Hospital, Trondheim, Norway; q Center for Neurotauma, MultiOmic & Biomarkers, Department of Neurobiology, Neuroscience Institute, Morehouse School of Medicine, Atlanta, USA; r Örebro University, School of Medical Sciences, Örebro, Sweden; s Department of Neurosurgery, Antwerp University Hospital, Edegem, Belgium; t Department of Translational Neurosciences, Faculty of Medicine and Health Science, University of Antwerp, Edegem, Belgium; u Department of Biomedical Data Sciences, Leiden University Medical Center, Leiden, the Netherlands

**Keywords:** Traumatic brain injury, Concussion, Imaging, Biomarkers, Prognostication, Outcome

## Abstract

**Background:**

Even patients with normal computed tomography (CT) head imaging may experience persistent symptoms for months to years after mild traumatic brain injury (mTBI). There is currently no good way to predict recovery and triage patients who may benefit from early follow-up and targeted intervention. We aimed to assess if existing prognostic models can be improved by serum biomarkers or diffusion tensor imaging metrics (DTI) from MRI, and if serum biomarkers can identify patients for DTI.

**Methods:**

We included 1025 patients aged >18 years with a Glasgow Coma Score >12 and normal CT from the Collaborative European NeuroTrauma Effectiveness Research in Traumatic Brain Injury (CENTER-TBI) study which recruited between December 19,2014 and December 17, 2017 (NCT02210221). Biomarkers (GFAP, NFL, S100B) were obtained at a median of 8.8 h (Q1–Q3 4.2–16.7) and DTI at 13 days (3–19) after injury. DTI metrics were available in 153 patients for 48 white matter tracts (ICBM-DTI-81 atlas). Incomplete recovery at three months was defined as an extended Glasgow Outcome Scale score <8. Existing prognostic models were fitted with and without biomarkers, or with and without DTI, and internally validated using bootstrapping.

**Findings:**

385 (38%) patients had incomplete recovery. Adding biomarkers did not improve performance beyond the best existing clinical prognostic model [optimism-corrected AUC 0.69 (95% CI 0.65–0.72) and R^2^ 17% (11–22)]. Adding DTI metrics significantly enhanced all models [best optimism-corrected AUC 0.82 (0.79–0.85) and R^2^ 75% (39–100)]. The top three prognostic tracts were the left posterior thalamic radiation, left superior cerebellar peduncle and right uncinate fasciculus. Serum biomarkers could have avoided 1 in 5 DTI scans, with GFAP <12 h and NFL 12–24 h from injury performing best.

**Interpretation:**

DTI substantially improved existing prognostic models for functional outcome in patients with mTBI and a normal CT, and biomarkers could help select patients for MRI. If validated, DTI could allow for targeted follow-up and enrichment of clinical trials of early interventions to improve outcome.

**Funding:**

EU Seventh Framework Programme, 10.13039/501100007731Hannelore Kohl Stiftung, 10.13039/100018727One Mind, 10.13039/100009006Integra LifeSciences, NeuroTrauma Sciences.

**Research in context**

**Evidence before this study**

We searched PubMed from its inception to December 4th, 2023. To identify studies predicting incomplete recovery after mTBI we used the search terms “mild traumatic brain injury” OR “concussion”; AND “prognosis” OR “prediction”; AND “Glasgow Outcome Scale”. As a quality criterion we only considered studies where models had been internally or externally validated. To find studies on the association between incomplete recovery and diffusion tensor imaging (DTI), we used the search terms “mild traumatic brain injury” OR “concussion”; AND “diffusion tensor imaging”. For studies on the role of biomarkers we searched “mild traumatic brain injury” OR “concussion”; AND the names of six biomarkers combined by OR: S100 calcium-binding protein B (S100B), glial fibrillary acidic protein (GFAP), ubiquitin C-terminal hydrolase L1 (UCH-L1), neuron-specific enolase (NSE), neurofilament protein-light (NFL), and total tau (t-tau).

The search identified five models using clinical variables to predict incomplete recovery from three formal prognostic studies, all using mixed mTBI cohorts with and without CT abnormalities. After internal validation, all models achieved areas under the curve (AUCs) ranging from 0.69 to 0.79 on these mixed cohorts. None of the models used diffusion tensor imaging (DTI) or any other magnetic resonance imaging (MRI) data as predictors. Evidence from two large multi-center cohort studies suggests an association between incomplete recovery and abnormalities identified on DTI within one month of injury. The predictive value of DTI, however, has not been tested in a formal prognostic study design.

Previous studies have demonstrated an incremental value of serum biomarkers over clinical characteristics when predicting functional recovery after TBI. However, this value was lower in mTBI and, where tested, even more so in the subgroup of patients with a normal CT. It remains unclear whether DTI can provide a greater incremental benefit than biomarkers for prognostication in mTBI. Previous demonstration of association between serum biomarkers and MRI abnormalities in CT-negative patients suggests that if DTI had greater prognostic value than serum biomarkers, serum biomarkers might still be used to select patients for DTI. While this has been explored in more severe TBI, it has not been formally studied in mild TBI. The results of these studies highlighted GFAP, S100B and NFL as the top three biomarkers for the prediction of incomplete recovery and MRI findings in patients with mTBI and a normal CT.

**Added value of this study**

To the authors knowledge, this is the first formal prognostic study to assess the value of DTI in the context of patients with mTBI and a normal CT. The findings validated that some existing models are improved by the addition of S100B (but not NFL or GFAP). However, none of the S100B-containing models outperformed the best reference model, suggesting that none of these serum biomarkers add value in CT-negative mTBI if other prognostic features are taken into account. The findings also demonstrate that DTI (mean diffusivity and fractional anisotropy in white matter tracts) substantially improved existing prognostic models, with areas under the curve >0.8 and a R^2^ >70%. The top three white matter tracts where abnormal DTI metrics were prognostic included the left posterior thalamic radiation, left superior cerebellar peduncle, and right uncinate fasciculus. Using serum biomarkers to select patients for MRI could have avoided 1 in 5 scans, with GFAP performing best <12 h and NFL best 12–24 h from injury (S100B was less specific at both time points).

**Implications of all the available evidence**

Findings of this formal prognostic study, supported by previous association studies, suggest that DTI can predict incomplete recovery in patients with mTBI and a normal CT, and is the best currently available prognostic tool for this population, outperforming serum biomarkers. Such a tool could allow for targeted follow-up and enrichment of clinical trials of early interventions to improve outcome. Prior to implementation these findings should be externally validated in a separate study cohort.

## Introduction

Affecting 50–60 million new patients every year, traumatic brain injury (TBI) is the most common neurological disorder worldwide.^1^ While approximately 90% of all TBI cases are classified as “mild”, this is a misnomer, since ∼20–50% of mild TBI (mTBI) patients experience persistent problems which may last months to years after injury.^2^, ^3^, ^4^, ^5^, ^6^ While traumatic lesion on X-ray computed tomography (CT) may predict poorer outcomes,^7^ less than 10% of patients who are scanned after mTBI have an abnormal CT, and high rates of incomplete recovery occur even in those with normal CT head scans.^5^ Our failure to identify such individuals with conventional imaging means those with persisting problems often fail to receive the ongoing care they need to maximise chance of good outcomes.^8^^,^^9^ There is a clear need to identify patients with mTBI with normal CT head scans who are at high risk of incomplete recovery, and this was a key research recommendation of the Lancet Neurology Commission for TBI 2022.^1^

Few predictive models for outcome after mTBI using clinically available features have undergone internal or external validation. All of these were developed on mTBI cohorts where a proportion of the patients had CT lesions present, and indeed in a higher proportion of lesions than would be expected (15, 18 and 46% respectively).^2^^,^^10^^,^^11^ It remains unclear how well these models would perform in an exclusively CT-negative mTBI cohort, and if they could be improved by the addition of two investigations that hold particular promise for aiding prognostication; serum protein biomarkers of brain injury,^12^^,^^13^ or diffusion tensor magnetic resonance imaging (DTI).^14^^,^^15^

The incremental prognostic value of serum protein biomarkers has been tested in large multi-centre studies, but not in the context of all of the clinically based reference models mentioned earlier.^12^^,^^13^ The US-based TRACK-TBI study showed that, for patients with mTBI, both glial fibrillary acidic protein (GFAP) and ubiquitin C-terminal hydrolase L1 (UCH-L1) improve the UPFRONT-PLUS model equally.^12^ Whilst there was no sub-group analysis of patients with mTBI and a normal CT, the TRACK-TBI consortium demonstrated that serum GFAP concentrations were associated with traumatic lesions on MRI in CT-negative patients with mTBI.^16^ CENTER-TBI collaborators tested the incremental benefit of six serum biomarkers individually and in combination, and when added to a bespoke model using clinical variables.^13^ A subgroup analysis of patients with mTBI and a normal CT showed that only the calcium-binding protein S100B improved the model. These studies highlight GFAP and S100B as the top two prognostic proteins in the CT-negative mTBI population. However, the final prognostic models using GFAP for all cases of mTBI (TRACK-TBI study) and S100B for CT-negative cases of mTBI (CENTER-TBI study) achieved areas under the curve of 0.69 and 0.66, respectively, suggesting that there is room for improvement (for example by using DTI.)

Diffusion tensor imaging (DTI), a magnetic resonance based neuroimaging technique to evaluate microstructural integrity, has been associated with outcome after mTBI in multiple studies, especially when performed soon after injury.^14^^,^^15^^,^^17^, ^18^, ^19^, ^20^ However, these studies all included 10–28% of patients with CT abnormalities; and subgroup analyses in two of these studies found that associations between DTI and outcome were attenuated when CT-positive patients were excluded.^17^^,^^18^ Furthermore, these studies reported on associations between imaging and outcome, and did not address a formal prognostic analysis (using internal or external validation) to assess the predictive value of DTI. Whilst both blood biomarkers and DTI may be useful for prognostication in the CT-negative mTBI population, their relative sensitivities and specificities remain unclear. Although DTI provides a very specific marker of microstructural disruption, it is expensive and less accessible in the acute setting. Given that GFAP and S100B are sensitive markers of TBI, and the axonal marker (neurofilament light; NFL) is thought to reflect traumatic axonal injury,^21^ it would be useful to explore whether these biomarkers can identify (or at least enrich) populations of patients who are more likely to show DTI abnormalities.

The objectives of the present study were therefore:

1. To validate the incremental prognostic value of serum GFAP, NFL and S100B when added to existing models predicting incomplete recovery in patients with mTBI and a normal CT.
2. To test, for the first time in a formal prognostic study adhering to TRIPOD Guidelines,^22^ the incremental prognostic value of DTI when added to existing models predicting incomplete recovery in patients with mTBI and a normal CT.
3. If DTI showed incremental prognostic value, to explore if serum GFAP, S100B or NFL could serve as a screening test to reduce the number of DTI scans required.

## Methods

### Study design and participants

We selected patients from the Collaborative European NeuroTrauma Effectiveness Research in Traumatic Brain Injury (CENTER-TBI) study (Supplementary Figure S1). This was a prospective longitudinal multinational cohort study which recruited from 19th December 2014 to 17th December 2017 at centers in Europe and Israel.^23^ Within the core cohort study was a planned MRI sub-study of 15 sites where recruited patients were invited to attend 3 T MRI within 72 h, ∼2–3 weeks, 3 months (ER stratum), 6 months (admission and ICU strata), 12 months and 24 months. CENTER-TBI was registered with ClinicalTrials.gov (NCT02210221). Informed written or oral consent by patients or legal representatives was obtained according to local regulation. The list of sites, ethics committees, approval numbers and approval dates can be found at: https://www.center-tbi.eu/project/ethical-approval. Clinical data was accessed via the Neurobot platform (RRID/SCR_017004, core data, version 3.0).

Inclusion criteria for the current analysis were GCS 13–15 on presentation and a normal acute CT. For the analysis assessing the prognostic value of biomarkers patients must have been aged ≥18 years and have biomarkers sampled within 24 h of injury. For the analysis assessing the prognostic value of DTI, patients must have been aged 20–70 years (to align the age range available in the control cohort ± 2 years, Supplementary Figure S2) and had MRI within 31 days of injury. The control cohort consisted of 157 healthy volunteers who were scanned using the same scanners and imaging protocols as the patients.

### Procedures

#### Image acquisition, reporting and processing

CT images were acquired using local site protocols. DTI was acquired on 17 different 3.0 T machines across 15 sites from three vendors: GE Systems (DISCOVERY MR750 and DISCOVERY MR750w models), Philips (Ingenia and Achieva models), and Siemens (Skyra, Prisma, Trio and Verio models). DTI protocols across CENTER-TBI were pre-agreed and included 32 directions with a b-value of 1000 s/mm^2^ and 2 mm^2^ isotropic voxels.^24^ Full protocols are available online (https://www.center-tbi.eu/project/mri-study-protocols).

CT and MR images were reported centrally using common data elements.^25^ All images were visually inspected to check for artefacts. Diffusion weighted images (DWI) were processed by a bespoke pipeline.^14^ Images were corrected for noise, field inhomogeneities and artefacts (Gibbs ringing, eddy current and motion induced).^14^ The diffusion tensors were calculated using weighted-least squares in FSL (https://fsl.fmrib.ox.ac.uk/fsl/) to create maps of mean diffusivity (MD) and fractional anisotropy (FA). These were then non-linearly registered to the JHU ICBM-DTI-81 atlas^26^ using ANTS^27^ to obtain mean values of MD and FA for all 48 individual white matter tracts.

#### Image post-processing steps

To remove machine differences, diffusion data was harmonized using the Combat algorithm which has been validated for use on DTI data.^28^^,^^29^ Age, (Age)^2^, Sex, Time since injury, GCS, and the presence/absence of any reported traumatic MR abnormality were used as covariates to preserve the biological variance associated with them.

White matter diffusion metrics change throughout the human lifespan, so they require age-correction in order to compare younger and older patients within the same study.^30^ However, the exact shape of this trajectory curve appears to differ by white matter tract, so any age-correction needs to be tract specific.^31^ We therefore adapted a “detrending” method previously validated in volumetric dementia imaging data.^32^ Healthy control data was used to estimate the normal ageing trajectory of each tract using a linear regression model with FA or MD as the dependent variable and Age and (Age)^2^ (as orthogonal polynomials) as the independent variables. The regression coefficients were then used to adjust the patient data, as if all patients had been imaged at the age of 44, which was the median age of patients in the DTI cohort. Take for example a patient aged 20 years old with an uncorrected FA value *f*. From the model fit to the controls we know that the normal difference in FA between a 20-year-old and a 44-year-old is *d*. The age-corrected FA value for our patient would be *f + d*.

#### Serum biomarker data

Serum for biomarkers was obtained within 24 h of injury and at ∼2 weeks after injury, and processed as previously described.^33^ In brief, samples were centrifuged within 60 min and stored at −80 °C and did not undergo further analysis until after recruitment closure. Analysis for S100B was performed at the University of Pecs, Hungary, using an electrochemiluminescence immunoassay kit (Elecsys S100 assay) run on the e 602 module of Cobas 8000 modular analyzer (Roche Diagnostics, Mannheim, Germany). Analysis for GFAP and NFL occurred at the University of Florida, USA, using a Single Molecule Arrays (SiMoA) based assay on the SR-X benchtop assay platform (Quanterix Corp., Lexington, MA).^33^

#### Outcome

Outcome was assessed at 3 months (76–125 days) using the extended Glasgow Outcome Score (GOSE) which was dichotomized into complete recovery (GOSE = 8) and incomplete recovery (GOSE <8).^34^ The GOSE is commonly used as the primary outcome measure in TBI studies. It reflects new injury-related dependence or difficulties (including worsening of pre-existing problems) encompassing the major areas of daily functioning. Changes in function were counted regardless of whether they resulted from TBI or other injury related factors including extracranial injury. Missing values for GOSE were centrally imputed by the CENTER-TBI data curation team (Supplementary Methods 1).

#### Reference models using clinical variables

To understand the current best prognostic models available for mTBI we conducted a literature review for existing models predicting incomplete recovery after mTBI. We searched PubMed from its inception to December 4th, 2023. To identify studies predicting incomplete recovery after mTBI we used the search terms “mild traumatic brain injury” OR “concussion”; AND “prognosis” OR “prediction”; AND “Glasgow Outcome Scale”. As a quality criterion we only considered studies where models had been internally or externally validated.

This search identified five models from three studies. The UPFRONT study generated two models–one using predictors collected in the emergency department only (UPFRONT-ED) and one using additional data from a psychological assessment at two weeks post-injury also (UPFRONT-PLUS).^2^^,^^10^ The Head Injury Serum Markers for Assessing Response to Trauma (HeadSMART) study model relies on acute mTBI symptoms as predictors.^11^ The CENTER-TBI study developed a range of models which, unlike previous models, included the injury severity score (ISS), thereby capturing some of the extra-cranial injury burden.^35^ Their best performing model will be referred to as CENTER-PLUS as it includes a psychological assessment at 2–3 weeks.^35^ The next best model not reliant on CT abnormalities (henceforth the CENTER-ED model), is based on post-concussion symptoms in the ED.^35^ Table 1 summarises these five existing prognostic models using clinical variables^2^^,^^11^^,^^35^ which were used as reference models to assess if the addition of serum biomarkers/DTI would provide incremental value.

**Table 1:** Existing models predicting incomplete recovery after mTBI.

Model name	Pre-injury variables	Injury variables	Two week variables
UPFRONT-ED	Age Sex Education level Prior mental health problems Age∗Education level (interaction)	Alcohol intoxication Neck pain GCS PTA ≥1 h	–
UPFRONT-PLUS	Education level Prior mental health problems	Alcohol intoxication Neck pain GCS PTA ≥1 h	Anxiety Depression Concussion symptom score Avoidant coping style Passive coping style
HeadSMART	Age Prior depression,	Positive head CT Moderate-severe headache Mild or worse difficulty concentrating Mild or worse photophobia	–
CENTER-ED	Age Sex Prior mental health problems Pre-injury health (ASA)	ISS GCS Mechanism of injury Pupils Concussion symptoms score	–
CENTER-PLUS	Age Prior mental health problems	ISS GCS Mechanism of injury	Concussion symptom score Post-traumatic stress disorder

GCS, Glasgow Coma Score; PTA, Post-traumatic amnesia; ASA level, American Society of Anesthesiologists Physical Status; ISS, Injury severity score. We had to omit the variables for neck pain and coping styles and had to substitute prior depression for prior mental health problems, as the original variables were not (consistently) recorded in the CENTER-TBI database. The variable pupil was also excluded as unreactive pupils were almost never observed (n = 3) in our cohort.

### Statistical analysis

Statistical analysis was conducted in R 4.3.1 (R Project for Statistical Computing). The statistical code is freely available at https://github.com/DrSophieRichter/DTImTBI. The association between visible traumatic abnormalities on MRI and an incomplete recovery was calculated using odds ratios. Unless otherwise indicated, data are presented as mean (95% confidence interval). Statistical tests were two-tailed, and p-values were considered significant if <0.05 after adjustment for multiple comparisons using the Benjamini-Hochberg method (applied per table).

Outcome data were missing in <18% and <3% of patients in the larger serum biomarker and smaller DTI cohorts, respectively. Missing data for acute variables ranged from 0–17% to 0–12%, respectively. Variables collected at two weeks were missing for 62–63% and 25–27% of patients, respectively. This may reflect the fact that patients who returned for DTI at 2 weeks post-injury could then also be interviewed about their symptoms. Therefore, reference models using two-week data were only used in the DTI cohort, not the larger biomarker cohort. Missing data was handled under the missing at random (MAR) assumption using multiple imputation as implemented in the R mice package. We used the MAR assumption according to the guidance on management of missing data in studies of TBI which was supported by the patients with and without missing data being similar in the most characteristics except for educational level which tended to be higher in patients with complete data (Supplementary Table S1).^36^ Ten imputed datasets were generated, and all subsequent analyses performed first within each imputed dataset before pooling results using Rubin's rules. To assess the approach taken for handling missing data sensitivity analyses were performed using best (assumption of complete recovery) and worst (assumption of incomplete recovery) case-analysis.

Given prior studies have found an association between NFL and DTI values at later timepoints correlations between whole brain MD and FA with NFL (on a logarithmic scale) were assessed using Pearson correlation coefficient.^37^^,^^38^ To identify the most prognostic of the 48 white matter tracts we used lasso logistic regression (see Supplementary Methods 2 for further details). “Incomplete recovery” was the outcome variable, the 96 DTI variables (FA and MD each for 48 tracts) were candidate predictor variables. The FA and MD values were age-corrected (as described above), centred and scaled (i.e., z-scored). The penalty factor lambda which would minimize the mean prediction error was identified through 10 × 10 cross-validation. The resulting model would have a reduced number of DTI variables—only those with the greatest prognostic value (see results section). We used this model to calculate for each patient the odds of an incomplete recovery i.e., the odds of an incomplete recovery based on DTI information alone (the “DTI score”). To assess the robustness of the DTI age-correction method described above a sensitivity analysis was performed using DTI data not corrected for age.

To understand if serum biomarkers/DTI could improve the reference models, we fitted the reference models with and without the serum biomarkers/DTI score. Because reference models had to be refitted to our study cohort (i.e., recalibrated with coefficients being updated), their model performance here would differ from that reported on different study cohorts in the literature. Model performance was measured using the area under the receiver operating characteristic curve (AUC), the Cox calibration intercept and a slope, the Nagelkerke R^2^, sensitivity, specificity, and positive and negative predictive values. Two nested models (the same model with versus without the DTI score) were compared using a likelihood ratio test.^39^ To provide realistic estimates of how well the models would perform on new unseen patient data, we performed internal validation of all model metrics using 200 bootstrap samples within each of 10 imputed datasets.

To understand if biomarkers could be used to select patients for DTI, we performed an exploratory analysis in those patients with DTI and biomarkers (NFL, GFAP and S100B). For the primary analysis we set a minimum sensitivity of 0.90 and chose a threshold concentration for each biomarker that would yield the highest specificity using the R package OptimalCutpoints. As a sensitivity analysis we also tested minimum sensitivities of 1.00, 0.95 and 0.80.

Results were reported as per TRIPOD for prognostic studies.^22^

### Role of the funding source

The funders of the study had no role in study design, data collection, data analysis, data interpretation, or writing of the report.

## Results

### Role of serum biomarkers for prognosis

Among 1025 patients with biomarkers, aged 18–93 (median 48), 656 (64%) male, 385 (38%) had an incomplete recovery (Supplementary Table S2). On this cohort, the CENTER-ED model performed best in isolation, with an AUC of 0.69 (0.65–0.72) and R^2^ of 17% (11–22) (Table 2). GFAP and NFL did not improve any reference models (Supplementary Tables S3 and S4). S100B improved some reference models but none exceeded the performance of the CENTER-ED model without biomarkers (Table 2). Using all three biomarkers together the results are almost identical to those of S100B alone supporting the conclusion that GFAP and NFL do not improve model performance (Supplementary Table S5). Model coefficients are given in Supplementary Table S6. The worst-case sensitivity analyses found there was no benefit of adding GFAP, a marginal benefit of adding NFL for some models (except for the best performing model) and marginal improvement of all models when adding S100B (Supplementary Tables S7–S10). The best-case analysis showed no benefit adding GFAP, no benefit adding NFL and marginal improvement to all models when adding S100B (Supplementary Tables S11–S14). A sensitivity analysis of NFL timing performed with and without subacute NFL found no improvement in model performance (Supplementary Table S15).

**Table 2:** Clinical model performance with and without S100B.

Metric	S100B only	UPFRONT-ED		HeadSMART		CENTER-ED	
		w/o S100B	with S100B	w/o S100B	with S100B	w/o S100B	with S100B
Area under the curve	0.57 (0.53–0.61)	0.57 (0.53–0.61)	0.60 (0.56–0.64)	0.58 (0.54–0.62)	0.62 (0.58–0.66)	0.69 (0.65–0.72)	0.69 (0.66–0.73)
Variation explained (%)	2 (0–5)	5 (1–8)	7 (3–11)	4 (1–8)	7 (3–11)	17 (11–22)	17 (12–23)
Sensitivity	0.13 (0.03–0.24)	0.28 (0.16–0.40)	0.33 (0.23–0.44)	0.33 (0.20–0.45)	0.36 (0.25–0.48)	0.48 (0.40–0.56)	0.48 (0.40–0.57)
Specificity	0.93 (0.87–0.98)	0.82 (0.73–0.90)	0.80 (0.73–0.88)	0.81 (0.73–0.88)	0.79 (0.73–0.86)	0.77 (0.72–0.83)	0.78 (0.72–0.83)
Positive predictive value	0.60 (0.50–0.70)	0.56 (0.50–0.62)	0.58 (0.53–0.64)	0.58 (0.51–0.65)	0.59 (0.53–0.65)	0.64 (0.58–0.69)	0.64 (0.59–0.69)
Negative predictive value	0.57 (0.54–0.60)	0.58 (0.54–0.61)	0.59 (0.56–0.63)	0.59 (0.56–0.63)	0.60 (0.57–0.64)	0.65 (0.61–0.68)	0.65 (0.61–0.68)
Likelihood ratio test p-value			<0.001		<0.001		0.056

All models are predicting incomplete recovery (extended Glasgow Outcome Scale score <8) at 3 months in patients with a mild traumatic brain injury and a normal CT. Numbers are given as means (95% confidence interval). All numbers have been corrected for optimism using bootstrapping. Note that the incremental value of biomarkers was assessed in three reference models while the incremental value of DTI was assessed on five reference models because two reference models required data obtained 2-weeks post-injury which was only consistently available in the smaller DTI cohort. An incremental prognostic effect was seen with S100B in some base models, as shown here. No improvement in prognostic efficiency was seen with addition of GFAP or NFL values in any of the models (see Supplemental Tables S2 and S3).

### Role of DTI for prognosis

MRI was available for 153 patients (aged 20–70 (median 44) years, 71% male) and 157 controls (aged 21–68 (median 39) years, 57% male) (Supplementary Figure S1, Supplementary Tables S1 and S16). At 3 months, 70 (46%) patients had incomplete recovery. The MRI was obtained at a median of 13 (Q1–Q3 3–19) days, i.e., within 3 days, within 4–14 days and within 15–31 days in 50 (33%), 35 (23%) and 68 (44%) of patients, respectively. Traumatic abnormalities were visible on MRI in 38 (25%) patients but their association with outcome did not reach statistical significance (Supplementary Table S17).

There was a weak negative association between whole brain white matter FA and NFL concentration consistent with poorer white matter integrity being associated with higher NFL levels (Supplementary Figure S3). This association disappeared when adjusted for age. There was no associated between MD and NFL concentrations. Using DTI, 21 of 48 white matter tracts, predominantly from the left hemisphere, were identified as prognostically relevant (Table 3, Supplementary Figure S4). The top three prognostic tracts included the left superior cerebellar peduncle, the right uncinate fasciculus, and the left posterior thalamic radiation.

**Table 3:** White matter tracts of high prognostic value identified by lasso regression.

White matter region		Frequency of selection		
Region	Hemisphere	FA freq	MD freq	Mean freq
Superior cerebellar peduncle	left	0.94	0.91	0.92
Uncinate fasciculus	right	0.83	0.94	0.88
Posterior thalamic radiation	left	0.96	0.78	0.87
Fornix/Stria terminalis	left	0.79	0.87	0.83
Retrolenticular part of internal capsule	left	0.61	0.88	0.74
Tapetum	left	0.78	0.58	0.68
Genu of corpus callosum	commissural	0.55	0.67	0.61
Cingulum (hippocampus)	left	0.91	–	–
Sagittal stratum	left	–	0.91	–
Pontine crossing tract	commissural	0.90	–	–
Corticospinal tract	right	–	0.87	–
Cingulum (hippocampus)	right	–	0.78	–
Middle cerebellar peduncle	commissural	–	0.77	–
Medial lemniscus	right	0.75	–	–
Inferior cerebellar peduncle	left	–	0.72	–
Body of corpus callosum	commissural	–	0.67	–
Splenium of corpus callosum	commissural	–	0.66	–
Superior fronto-occipital fasciculus	left	–	0.62	–
Posterior limb of internal capsule	left	0.54	–	–
Posterior thalamic radiation	right	–	0.49	–
Superior fronto-occipital fasciculus	right	0.38	–	–

The table shows those tracts from JHU ICBM-DTI-81 atlas which were identified as prognostic based on lasso regression. Tracts are listed in order of prognostic value determined by the frequency of selection, i.e., the proportion of times the tract was selected as prognostically relevant in the 2000 runs that comprised the analysis (200 bootstrap samples x 10 multiply imputed datasets). FA freq = how often the tract was selected for its fractional anisotropy (FA) value, MD freq = how often the tract was selected for its mean diffusivity (MD) value, Mean freq = average of FA and MD.

**Fig. 1:**
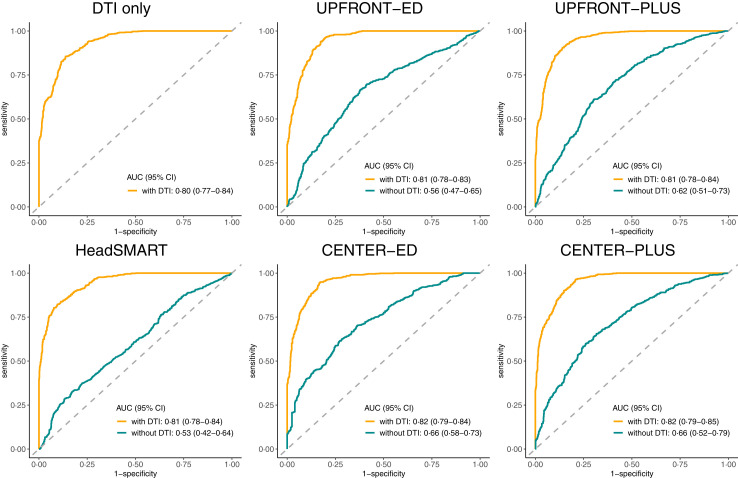
**Receiver operating characteristics curves for models with (yellow line) and without (green line) diffusion tensor imaging (DTI)**. AUC (95% CI), Area under the curve (95% confidence interval).

**Fig. 2:**
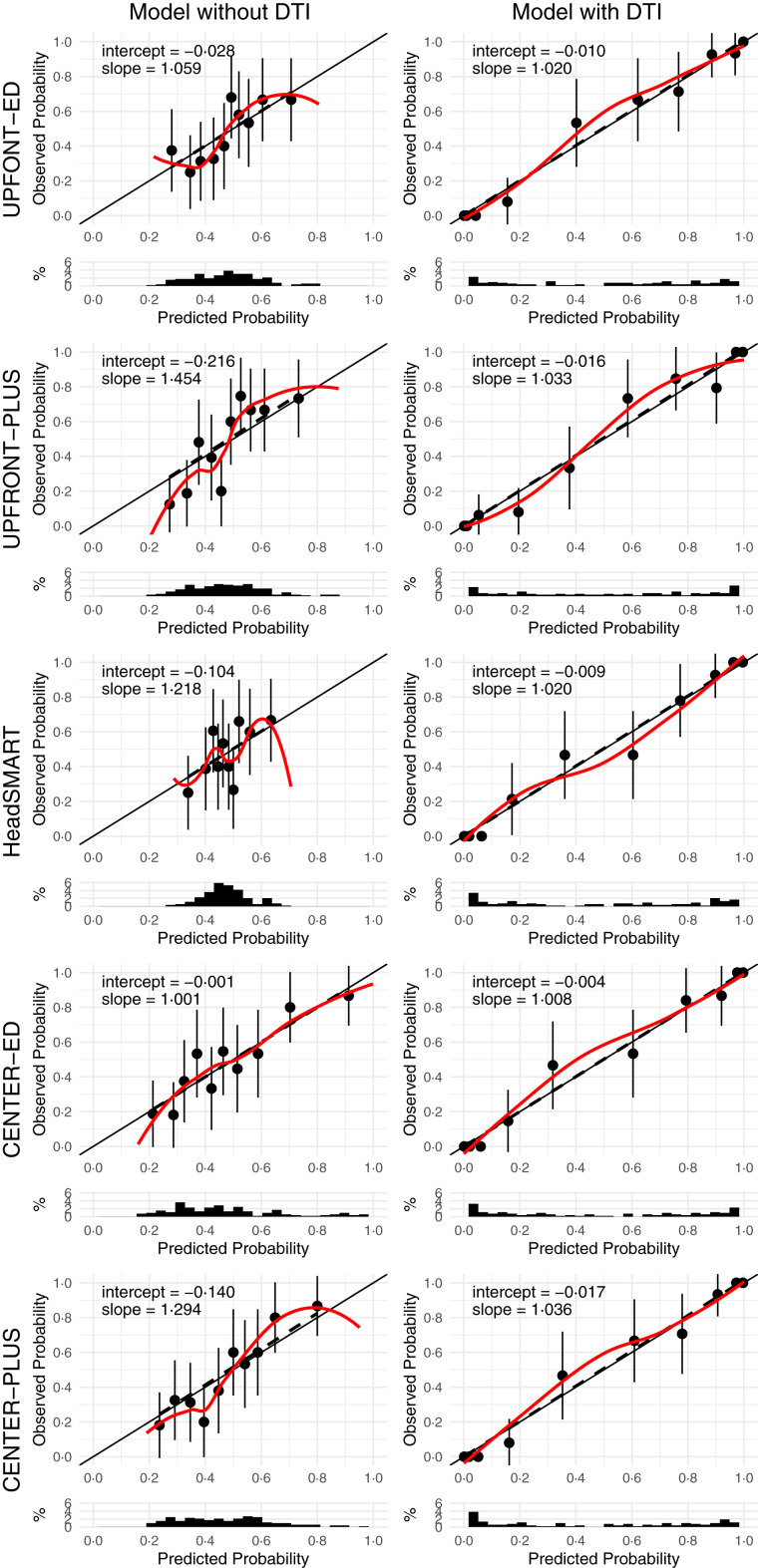
**Calibration plots for models with and without diffusion tensor imaging (DTI)**. Each prognostic model is presented on a new row. Each row shows the calibration plot of the same model without (left-hand panel) and with (right-hand panel) DTI as a predictor. Dots with bars = mean predicted probability of unfavorable outcome with its 95% confidence interval. Solid line = calibration line that should be followed by a perfectly calibrated model. Dashed line = calibration line of the actual model, fitted using linear regression. Coloured line = calibration line of the actual model, fitted using locally estimated scatterplot smoothing.

The addition of DTI data in the form of a DTI score (see methods) significantly increased the AUC of all models to >0.80 and R^2^ to >70%, p < 0.001 (Table 4, Fig. 1). Fig. 2 illustrates that model calibration was adequate for all models with and without DTI, but that DTI improved discrimination of patients with complete versus incomplete recovery. Model coefficients are given in Supplementary Table S18. The sensitivity analysis using DTI data not corrected for age found similar results to the age-adjusted data (Supplementary Table S19). The sensitivity analysis of best and worst case scenarios showed that adding DTI substantially improved all models (Supplementary Tables S20–S21).

**Table 4:** Performance of prognostic models with and without diffusion tensor imaging.

Metric	DTI only	UPFRONT-ED		UPFRONT-PLUS		HeadSMART		CENTER-ED		CENTER-PLUS	
		w/o DTI	with DTI	w/o DTI	with DTI	w/o DTI	with DTI	w/o DTI	with DTI	w/o DTI	with DTI
Area under the curve	0.80 (0.77–0.84)	0.56 (0.47–0.65)	0.81 (0.78–0.83)	0.62 (0.51–0.73)	0.81 (0.78–0.84)	0.53 (0.42–0.64)	0.81 (0.78–0.84)	0.66 (0.58–0.73)	0.82 (0.79–0.84)	0.66 (0.52–0.79)	0.82 (0.79–0.85)
R^2^(%)	70 (53–86)	9 (−4 to 22)	75 (2–100)	14 (−1 to 29)	74 (−5 to 100)	4 (−5 to 13)	73 (−100 to 100)	22 (8–36)	75 (−100 to 100)	19 (0–38)	75 (39–100)
Sensitivity	0.69 (0.60–0.78)	0.49 (0.26–0.72)	0.73 (0.65–0.80)	0.50 (0.26–0.74)	0.73 (0.65–0.81)	0.37 (0.03–0.71)	0.71 (0.63–0.80)	0.49 (0.32–0.66)	0.71 (0.61–0.80)	0.54 (0.33–0.75)	0.71 (0.63–0.79)
Specificity	0.71 (0.62–0.81)	0.63 (0.44–0.82)	0.72 (0.65–0.79)	0.68 (0.50–0.87)	0.73 (0.67–0.79)	0.66 (0.34–0.98)	0.70 (0.62–0.77)	0.72 (0.57–0.86)	0.75 (0.67–0.82)	0.70 (0.54–0.86)	0.73 (0.66–0.80)
Positive predictive value	0.69 (0.60–0.78)	0.54 (0.43–0.65)	0.70 (0.63–0.77)	0.59 (0.45–0.73)	0.71 (0.64–0.78)	0.51 (0.35–0.67)	0.67 (0.59–0.75)	0.62 (0.52–0.72)	0.72 (0.64–0.80)	0.62 (0.49–0.76)	0.70 (0.63–0.78)
Negative predictive value	0.72 (0.64–0.80)	0.57 (0.48–0.67)	0.75 (0.68–0.82)	0.60 (0.48–0.71)	0.75 (0.68–0.82)	0.53 (0.42–0.64)	0.73 (0.66–0.81)	0.61 (0.52–0.69)	0.74 (0.65–0.82)	0.63 (0.51–0.74)	0.74 (0.67–0.81)
Likelihood ratio test p-value			<0.001		<0.001		<0.001		<0.001		<0.001

All models are predicting incomplete recovery (extended Glasgow Outcome Scale score <8) at 3 months in patients with a mild traumatic brain injury and a normal CT. Numbers are given as means (95% confidence interval). All numbers have been corrected for optimism using bootstrapping. Note that the incremental value of biomarkers was assessed in three reference models while the incremental value of DTI was assessed on five reference models because two reference models required data obtained 2-weeks post-injury which was only consistently available in the smaller DTI cohort.

### Role of serum biomarkers to select patients for DTI

A subgroup of 108 patients within the DTI cohort had biomarkers sampled within 24 h (Supplementary Table S22). Biomarker thresholds were chosen to yield the highest possible specificity for the prediction of an incomplete recovery, given a minimum sensitivity of 0.90. If we had decided to only obtain DTI in patients with biomarker concentrations above the chosen threshold, then using serum biomarkers could have avoided 1 in 5 DTI scans, with GFAP performing best <12 h and NFL best 12–24 h from injury (Table 5). At the cutoff thresholds shown in this table <3% of patients would not have qualified for DTI even though they eventually did not recover completely. Results for different cutoff thresholds are shown in Supplementary Table S23. The results of the sensitivity analysis in the overlap cohort are presented in Supplementary Tables S24–S28. The results show that the best performing reference model (CENTER-ED) is improved marginally by S100B, reaching an AUC of 0.68 (0.61–0.76). In contrast, the same model is substantially improved by DTI, reaching an AUC of 0.83 (0.80–0.86). These results show that the conclusions drawn from the larger biomarker and DTI cohorts do also hold for the smaller overlap cohort.

**Table 5:** Using biomarkers to identify patients for DTI, using a minimum sensitivity of 0.9.

Sample	Time	N	Cutoff	Sensitivity	Specificity	Avoided MRIs	Unnecessary MRIs	Missed incomplete recoveries	Number needed to scan
GFAP	<12 h	63	0.13	0.92 (0.73–0.97)	0.30 (0.18–0.46)	13 (21%)	26 (41%)	2 (3%)	2.1
GFAP	12–24 h	44	0.09	0.95 (0.72–0.99)	0.22 (0.10–0.44)	6 (14%)	18 (41%)	1 (2%)	1.9
S100B	<12 h	63	0.04	1.00 (0.76–1.00)	0.11 (0.05–0.26)	4 (6%)	33 (52%)	0 (0%)	2.3
S100B	12–24 h	44	0.03	1.00 (0.72–1.00)	0.04 (0.01–0.26)	1 (2%)	22 (50%)	0 (0%)	2.1
NFL	<12 h	63	2.56	0.92 (0.73–0.97)	0.08 (0.03–0.23)	5 (8%)	34 (54%)	2 (3%)	2.5
NFL	12–24 h	44	5.41	0.90 (0.68–0.97)	0.35 (0.19–0.56)	10 (23%)	15 (34%)	2 (5%)	1.8

Biomarker cutoff levels were chosen assuming a minimum sensitivity of 0.90. Avoided MRIs refers to the number of patients which would not be scanned due to their biomarker level falling below the cutoff threshold and who would make a complete recovery (true negatives). Unnecessary MRIs refer to the number of patients who would have been scanned as their biomarker value exceeds the cutoff threshold but who would make a complete recovery (false positives). Missed incomplete recoveries refers to the number of patients who would not be scanned as their biomarker value falls below the cutoff threshold but who nonetheless would not recover completely (false negatives). Cutoff units are ng/ml (GFAP), μg/L (S100B) and pg/ml (NFL).

## Discussion

To our knowledge, this prognostic study of incomplete recovery after mTBI, is the first to assess the value of DTI and the first to focus specifically on patients with a normal CT. The findings showed that no biomarker containing model outperformed the best reference model based on clinical variables, suggesting that the studied serum biomarkers may not be useful in CT-negative mTBI, if other clinical characteristics are available. The findings also demonstrate that incorporation of FA and MD from DTI scans substantially improve existing prognostic models, with AUCs >0.8 and a R^2^ >70%. Based on subgroup analyses we show that acute concentrations of GFAP or NFL could potentially aid the patient selection for early DTI after mTBI.

Our analysis yielded a short-list of the most prognostic white matter tracts for functional outcome after mTBI. While both left and right sided structures, as well as commissural tracts were represented in this list, it was noteworthy that left sided structures were, in general, ranked more highly on this list. The predominance of left-sided tracts relevant to functional outcome after mTBI observed in our study has also been reported by Palacios et al.^15^ We concur with their speculation regarding the importance of the left hemisphere for language processing and right-hand actions. The top three tracts included the left superior cerebellar peduncle, the right uncinate fasciculus and the left posterior thalamic radiation. Potentially acceleration-deceleration forces may act more strongly on long association tracts and the delicate bridges of the superior cerebellar peduncles. Alternatively, these tracts hold particular functional importance.

The relevance of thalamo-cortical connections in general and the left posterior thalamic radiation in particular is consistent with previous studies demonstrating altered structural or functional thalamic connectivity in the acute-subacute phase of patients with mTBI and persistent symptoms.^18^^,^^40^ These might be explained by the role of thalamocortical connections in brain-wide information processing including attention, working memory, motor coordination, and sleep–wake cycles.^41^ Diffusion abnormalities in the superior cerebellar peduncle have previously been reported, although cerebellar structures in general have received less attention in DTI studies of mTBI.^42^ The superior cerebellar peduncles consist predominantly of efferent fibers from the cerebellum to the thalamus. Both the superior cerebellar peduncle and the posterior thalamic radiation are vital parts in the prefrontal-parietal-cerebellar network.^43^ According to the predictive brain state model, this network, with feed-forward timing from the cerebellum, is responsible for anticipating events, thereby targeting attention.^43^ A breakdown of this network due to white matter shearing, has been proposed to account for the attention and memory deficits observed after TBI.^43^ Although alterations in the right uncinate fasciculus have been observed in previous DTI studies of mTBI, its role in TBI recovery is less clear.^17^ Abnormalities in the tract has been implicated in the development of depression, anti-social behavior, and post-TBI post-traumatic stress disorder.^44^ Patients with an emotionally resilient mTBI phenotype have been found to have differences in DTI metrics to patients with a neuropsychiatrically distressed phenotype.^45^ Potentially it is a combination of biomechanical vulnerability to shear stress and a lack of functional reserve if damaged, that make certain tracts more prognostically important. Our finding that DTI is highly predictive alongside the lack of predictive power for subacute NFL is supportive of the hypothesis that DTI may be measuring pre-injury “reserve” as well as injury severity which has important implications for future preventative strategies of long-term sequelae. Based on previous association studies, we suspected that early DTI would improve clinical prediction models.^14^^,^^15^^,^^17^, ^18^, ^19^ However, we were surprised by the magnitude of improvement that the we observed - the addition of DTI to clinical models raised model performance to AUCs >0.8 and R^2^ values ≥70%. These results were robust during internal validation using bootstrapping, suggesting that if a new patient were to present to one of the CENTER-TBI sites and be scanned using the CENTER-TBI protocol, we could predict their probability of recovery with good accuracy. Having such a reliable prognostic model could allow us to stratify patients for clinical follow up or interventional trials within days of their injury (Supplementary Figure S5). Perhaps equally importantly, our findings provide a robust biological basis for incomplete recovery, which was previously unexplained or labelled as functional neurological disorder.^46^

Prediction of outcome for patients who have mTBI and a normal CT is particularly challenging and there are few studies which have focused on this cohort despite them being the majority of patients affected by mTBI. The best existing clinical prognostic models for mTBI were all derived on cohorts which included patients with traumatic lesions detectable on CT. We observed that these models (without DTI or biomarkers) performed worse in our cohort than in their derivation cohorts, even though the model developers performed the same internal validation procedure as we did.^2^^,^^11^^,^^35^ In part this may be due to the slight modifications we had to make to the models, as not all variables were recorded in the CENTER-TBI dataset. However, even in the validation paper by Mikolic et al., which used CENTER-TBI variables as we did, the AUC for the UPFRONT-PLUS model was 0.70 (95% CI 0.66–0.75), much higher than we observed.^10^ The poorer performance of the models is most likely due to the differences in case mix, i.e., that outcome prediction appears to be more difficult in patients with a normal CT than in those with CT abnormalities.

The findings that the tested blood biomarkers have little to no prognostic value over clinical parameters in patients with mTBI are consistent with prior studies where the prognostic value was greater in patients with a more severe burden of injury characterised by lower GCS and/or CT findings.^12^^,^^13^^,^^47^ Their potential use to help rationalise selection of patients for MRI provides important context for future use in clinical pathways. An improved understanding of how best to integrate all the information obtained from clinical, blood and imaging biomarkers is critical to enable more refined assessment which in turn may inform clinical trial design and lead to improved management and outcomes.

Whilst the prospective multi-centre design and formal prognostic methodology are major strengths of this paper, there are also limitations. Our study focused on patients with mTBI who presented to hospital and satisfied criteria for CT-scanning. Whether DTI shows equal prognostic value in patients on the even milder spectrum of TBI, i.e., those not qualifying for a CT or those not presenting to hospital, remains unclear. While the quantitative MRI analysis occurred after data collection was closed for patient safety the clinical sequences were available for clinical teams to review which may introduce treatment bias. In addition, our time window for DTI ranged from 0 to 31 days. We have previously shown that DTI changes are dynamic and more prognostic closer to the time of injury in a cohort of patients with mTBI without and without lesions on CT.^14^ Future studies should endeavor to use a more uniform and/or earlier timepoint. Only patients aged 20–70 years old were included to align our control population used to age-correct DTI metrics. Whether the prognostic value of DTI extends to children or older adults, especially those with pre-existing neurological disease, remains to be determined. Furthermore, not all patients who had serum biomarkers sampled also had DTI data available and vice versa, requiring us to use differently sized sub-cohorts from the CENTER-TBI study.

Our analysis using serum biomarkers to triage patients for DTI was based on a sub-sample of patients too small to fully control for difference in sample timing. The suggested threshold concentrations therefore need to be externally validated in a larger sample with a pre-defined sample time (or times) which could also consider the individual kinetic profiles of different serum biomarkers. No sites at the time of recruitment had access to point of care (POC) biomarker testing outside of S100B, and only two subjects in the MRI cohort had S100B levels obtained in the ER. At time of recruitment S100B was obtained only as decision support for CT and it is unlikely it caused any bias to the results obtained.^48^ Future studies should be wary of this potential source of bias as POC testing becomes more widespread.

Access to rehabilitation may confound prognostic studies. In the cohort with MRI only one was admitted to an inpatient rehabilitation facility and 24% were noted to have some form of outpatient therapy. The was mainly physiotherapy, with only one patient given cognitive therapy and five access to psychological services, and is consistent with the broader CENTER-TBI cohort,^49^ further emphasising the lack of rehabilitation resources offered to patients specific to mTBI. In addition, given that extracranial injury may also influence outcome, more complete characterisation in the acute phase and how much it contributes to long-term outcome may add important insights in future studies.^16^^,^^34^^,^^50^

Now that we have established a potential prognostic role for DTI future studies should assess if a new model can be developed that can predict GOSE on an ordinal scale and/or more refined outcome measures to capture the nuances of recovery after mTBI. In addition, in order to maximise the numbers for the analysis we did include patients from all strata and understanding whether there are different prognostic factors for different strata may be important for any future clinical implementation.

External validation of these results is required. In particular, some of the patients included may have been part of the derivation cohort for the CENTER-ED/CENTER-PLUS models, which may result in overestimation of the predictive power of the model. The analyses presented here are notable for finding a strong prediction signal from DTI despite being based on a multi-site study. This suggestions that DTI metrics may be sensitive prognostic biomarkers in this patient population. Implementation of comparable sequence acquisition between scanners needs to be recognised as a major barrier to the generalisation of DTI findings. Clinical implementation therefore will require collaboration between clinical and research institutions to choose similar MRI models and acquisition sequences alongside MRI manufacturers to synchronise their hardware and default settings is required.

This study showed that DTI can substantially improve existing prognostic models using clinical variables in patients with mTBI and a normal CT brain, and suggest that DTI is the is the best currently available prognostic tool for this population, outperforming serum biomarkers. Serum biomarkers may help to select those patients who may most benefit from the prognostic value of an MRI. A combination of clinical features, early biomarkers for MRI selection and DTI offer promise for the design of future clinical pathways which will help with appropriate stratification to ensure patients who require it are offered follow up and so potentially improve outcomes, and to inform the design of future clinical trials. Further studies are needed to reproduce these findings before adoption into clinical pathways.

## Contributors

SR, DM and VFJN conceived and designed this study. AM and DM were principal investigators for the CENTER-TBI project. GW, TD, OT, JP, AV, KG, AKH, MC, DM, VFJN planned the protocols for DTI acquisition. SW, EK, TD, JV, VFJN, DW and MC curated, reported and/or processed images from CT and/or MRI. EC, KW, AB were responsible for the storage and processing of serum biomarker samples. SR conducted the statistical analysis with oversight from ES and VFJN. SR wrote the first draft of the manuscript with input from VFJN. All authors helped to revise the manuscript. All authors had full access to all the data in the study and had final responsibility for the decision to submit for publication. SR and VFJN accessed and verified the data.

## Data sharing statement

Individual participant data will be available immediately after publication, conditional to an approved study proposal, with no end date. Data will be available to researchers who provide a methodologically sound study proposal that is approved by the management committee. Proposals can be submitted online at https://www.center-tbi.eu/data. A data access agreement is required, and all access must comply with regulatory restrictions imposed on CENTER-TBI.

## Declaration of interests

DM received personal fees from Lantmannen AB, GlaxoSmithKline plc, Calico Life Sciences LLC, PresSura Neuro, Integra Neurosciences, and NeuroTrauma Sciences, LLC; grants from GlaxoSmithKline plc; and a shared National Institutes of Health grant from Gryphon Bio Collaborators on a grant application outside the presented work. VFJN holds grants from Roche Pharmaceuticals for an analysis outside the presented work. AIRM declares personal fees from NeuroTrauma Sciences and Novartis and participated on the DSMB of PresSura Neuro during the conduct of the study. JP reports grant from Research council of Finland, during the conduct of the study. OT declares personal fees from NeuroTrauma Sciences and Abbott outside this work. KKW is a Co-Founder and share-holder of Gryphon Bio, Inc. (USA)—a CNS molecular diagnostic company.

## Appendix A Supplementary data

Supplement Part 1

Supplement Part 2

## References

[1] Maas A.I.R., Menon D.K., Manley G.T. (2022). Traumatic brain injury: progress and challenges in prevention, clinical care, and research. Lancet Neurol.

[2] van der Naalt J., Timmerman M.E., de Koning M.E. (2017). Early predictors of outcome after mild traumatic brain injury (UPFRONT): an observational cohort study. Lancet Neurol.

[3] Nelson L.D., Temkin N.R., Dikmen S. (2019). Recovery after mild traumatic brain injury in patients presenting to US level I Trauma centers. JAMA Neurol.

[4] Steyerberg E.W., Wiegers E., Sewalt C. (2019). Case-mix, care pathways, and outcomes in patients with traumatic brain injury in CENTER-TBI: a European prospective, multicentre, longitudinal, cohort study. Lancet Neurol.

[5] Madhok D.Y., Rodriguez R.M., Barber J. (2022). Outcomes in patients with mild traumatic brain injury without acute intracranial traumatic injury. JAMA Netw Open.

[6] Brett B.L., Temkin N., Barber J.K. (2023). Long-term multidomain patterns of change after traumatic brain injury: a TRACK-TBI long study. Neurology.

[7] MRC CRASH Trial Collaborators (2008). Predicting outcome after traumatic brain injury: practical prognostic models based on large cohort of international patients. BMJ.

[8] Foks K.A., Cnossen M.C., Dippel D.W.J. (2017). Management of mild traumatic brain injury at the emergency department and hospital admission in Europe: a survey of 71 neurotrauma centers participating in the CENTER-TBI study. J Neurotrauma.

[9] Seabury S.A., Gaudette É., Goldman D.P. (2018). Assessment of follow-up care after emergency department presentation for mild traumatic brain injury and concussion. JAMA Netw Open.

[10] Mikolic A., Polinder S., Steyerberg E.W. (2021). Prediction of global functional outcome and post-concussive symptoms after mild traumatic brain injury: external validation of prognostic models in the collaborative European NeuroTrauma effectiveness research in traumatic brain injury (CENTER-TBI) study. J Neurotrauma.

[11] Falk H., Bechtold K.T., Peters M.E. (2021). A prognostic model for predicting one-month outcomes among emergency department patients with mild traumatic brain injury and a presenting glasgow coma scale of fifteen. J Neurotrauma.

[12] Korley F.K., Jain S., Sun X. (2022). Prognostic value of day-of-injury plasma GFAP and UCH-L1 concentrations for predicting functional recovery after traumatic brain injury in patients from the US TRACK-TBI cohort: an observational cohort study. Lancet Neurol.

[13] Helmrich I.R.A.R., Czeiter E., Amrein K. (2022). Incremental prognostic value of acute serum biomarkers for functional outcome after traumatic brain injury (CENTER-TBI): an observational cohort study. Lancet Neurol.

[14] Richter S., Winzeck S., Kornaropoulos E.N. (2021). Neuroanatomical substrates and symptoms associated with magnetic resonance imaging of patients with mild traumatic brain injury. JAMA Netw Open.

[15] Palacios E.M., Yuh E.L., Mac Donald C.L. (2022). Diffusion tensor imaging reveals elevated diffusivity of white matter microstructure that is independently associated with long-term outcome after mild traumatic brain injury: a TRACK-TBI study. J Neurotrauma.

[16] Yue J.K., Yuh E.L., Korley F.K. (2019). Association between plasma GFAP concentrations and MRI abnormalities in patients with CT-negative traumatic brain injury in the TRACK-TBI cohort: a prospective multicentre study. Lancet Neurol.

[17] Yuh E.L., Cooper S.R., Mukherjee P. (2014). Diffusion tensor imaging for outcome prediction in mild traumatic brain injury: a TRACK-TBI study. J Neurotrauma.

[18] Stenberg J., Eikenes L., Moen K.G., Vik A., Haberg A.K., Skandsen T. (2021). Acute diffusion tensor and kurtosis imaging and outcome following mild traumatic brain injury. J Neurotrauma.

[19] Karlsen R.H., Einarsen C., Moe H.K. (2019). Diffusion kurtosis imaging in mild traumatic brain injury and postconcussional syndrome. J Neurosci Res.

[20] Bai L., Bai G., Wang S. (2020). Strategic white matter injury associated with long-term information processing speed deficits in mild traumatic brain injury. Hum Brain Mapp.

[21] Shahim P., Politis A., van der Merwe A. (2020). Neurofilament light as a biomarker in traumatic brain injury. Neurology.

[22] Collins G.S., Reitsma J.B., Altman D.G., Moons K.G. (2015). Transparent reporting of a multivariable prediction model for individual prognosis or diagnosis (TRIPOD). Ann Intern Med.

[23] Maas A.I., Menon D.K., Steyerberg E.W. (2015). Collaborative European NeuroTrauma effectiveness research in traumatic brain injury (CENTER-TBI): a prospective longitudinal observational study. Neurosurgery.

[24] Timmermans C., Smeets D., Verheyden J. (2019). Potential of a statistical approach for the standardization of multicenter diffusion tensor data: a phantom study. J Magn Reson Imaging.

[25] Vande Vyvere T., Wilms G., Claes L. (2019). Central versus local radiological reading of acute computed tomography characteristics in multi-center traumatic brain injury research. J Neurotrauma.

[26] Mori S.W.S., van Zijl P.C.M., Nagae-Poetscher L.M. (2005).

[27] Avants B.B., Tustison N.J., Song G., Cook P.A., Klein A., Gee J.C. (2011). A reproducible evaluation of ANTs similarity metric performance in brain image registration. Neuroimage.

[28] Richter S., Winzeck S., Correia M.M. (2022). Validation of cross-sectional and longitudinal ComBat harmonization methods for magnetic resonance imaging data on a travelling subject cohort. Neuroimage Rep.

[29] Fortin J.P., Parker D., Tunc B. (2017). Harmonization of multi-site diffusion tensor imaging data. Neuroimage.

[30] Westlye L.T., Walhovd K.B., Dale A.M. (2010). Life-span changes of the human brain white matter: diffusion tensor imaging (DTI) and volumetry. Cereb Cortex.

[31] Behler A., Kassubek J., Muller H.P. (2021). Age-related alterations in DTI metrics in the human brain-consequences for age correction. Front Aging Neurosci.

[32] Falahati F., Ferreira D., Soininen H. (2016). The effect of age correction on multivariate classification in Alzheimer's disease, with a focus on the characteristics of incorrectly and correctly classified subjects. Brain Topogr.

[33] Czeiter E., Amrein K., Gravesteijn B.Y. (2020). Blood biomarkers on admission in acute traumatic brain injury: relations to severity, CT findings and care path in the CENTER-TBI study. eBioMedicine.

[34] Wilson L., Boase K., Nelson L.D. (2021). A manual for the glasgow outcome scale-extended interview. J Neurotrauma.

[35] Mikolic A., Steyerberg E.W., Polinder S. (2023). Prognostic models for global functional outcome and post-concussion symptoms following mild traumatic brain injury: a collaborative European NeuroTrauma effectiveness research in traumatic brain injury (CENTER-TBI) study. J Neurotrauma.

[36] Richter S., Stevenson S., Newman T. (2019). Handling of missing outcome data in traumatic brain injury research: a systematic review. J Neurotrauma.

[37] Graham N.S.N., Zimmerman K.A., Moro F. (2021). Axonal marker neurofilament light predicts long-term outcomes and progressive neurodegeneration after traumatic brain injury. Sci Transl Med.

[38] Newcombe V.F.J., Ashton N.J., Posti J.P. (2022). Post-acute blood biomarkers and disease progression in traumatic brain injury. Brain.

[39] Vickers A.J., Cronin A.M., Begg C.B. (2011). One statistical test is sufficient for assessing new predictive markers. BMC Med Res Methodol.

[40] Woodrow R.E., Winzeck S., Luppi A.I. (2023). Acute thalamic connectivity precedes chronic post-concussive symptoms in mild traumatic brain injury. Brain.

[41] Shine J.M., Lewis L.D., Garrett D.D., Hwang K. (2023). The impact of the human thalamus on brain-wide information processing. Nat Rev Neurosci.

[42] Mac Donald C., Johnson A., Cooper D. (2013). Cerebellar white matter abnormalities following primary blast injury in US military personnel. PLoS One.

[43] Ghajar J., Ivry R.B., Cognitive, Neurobiological Research C (2008). The predictive brain state: timing deficiency in traumatic brain injury?. Neurorehabil Neural Repair.

[44] Williamson J.B., Heilman K.M., Porges E.C., Lamb D.G., Porges S.W. (2013). A possible mechanism for PTSD symptoms in patients with traumatic brain injury: central autonomic network disruption. Front Neuroeng.

[45] Cai L.T., Brett B.L., Palacios E.M. (2024). Emotional resilience predicts preserved white matter microstructure following mild traumatic brain injury. Biol Psychiatry Cogn Neurosci Neuroimaging.

[46] Hallett M., Aybek S., Dworetzky B.A., McWhirter L., Staab J.P., Stone J. (2022). Functional neurological disorder: new subtypes and shared mechanisms. Lancet Neurol.

[47] Whitehouse D.P., Monteiro M., Czeiter E. (2022). Relationship of admission blood proteomic biomarkers levels to lesion type and lesion burden in traumatic brain injury: a CENTER-TBI study. eBioMedicine.

[48] Unden L., Calcagnile O., Unden J., Reinstrup P., Bazarian J. (2015). Validation of the Scandinavian guidelines for initial management of minimal, mild and moderate traumatic brain injury in adults. BMC Med.

[49] Howe E.I., Zeldovich M., Andelic N. (2022). Rehabilitation and outcomes after complicated vs uncomplicated mild TBI: results from the CENTER-TBI study. BMC Health Serv Res.

[50] Carroll E.L., Manktelow A.E., Outtrim J.G. (2020). Influence of concomitant extracranial injury on functional and cognitive recovery from mild versus moderateto severe traumatic brain injury. J Head Trauma Rehabil.

