# Supplementary material for: Predicting recovery in patients with mild traumatic brain injury and a normal CT using serum biomarkers and diffusion tensor imaging (CENTER-TBI): an observational cohort study
Source: eClinicalMedicine. 2024 Aug 8;75:102751. doi: 10.1016/j.eclinm.2024.102751 (PMC11667275; doi:10.1016/j.eclinm.2024.102751)
Supplement: Supplement Part 1 [file mmc1.docx]

# Supplement

Predicting recovery in patients with mild traumatic brain injury and a normal CT using serum biomarkers and diffusion tensor imaging (CENTER-TBI): an observational cohort study

Sophie Richter; Stefan Winzeck,  Marta M. Correia; Endre Czeiter; Dan Whitehouse; Evgenios N. Kornaropoulos; Guy B. Williams; Jan Verheyden; Tilak Das;  Olli Tenovuo, Jussi Posti, Anne Vik, Kent Gøran Moen; Asta Kristine Håberg; Kevin Wang, Andras Buki, Andrew Maas, Ewout Steyerberg, David K. Menon; Virginia F. J. Newcombe; for the Collaborative European NeuroTrauma Effectiveness Research in Traumatic Brain Injury Magnetic Resonance Imaging (CENTER-TBI MRI) Substudy Participants and Investigators

**Contents**

Supplementary Methods 1. **Collection and central imputation of the GOSE variable at 3 months**

**Supplementary Methods 2. Explanation of the threshold used to choose the most prognostically significant DTI tracts**

**Supplementary Table 1. Comparison of patients with and without missing data**

Supplementary Table 2. Characteristics of included patients

Supplementary Table 3. Model performance with and without GFAP

Supplementary Table 4. Model performance with and without NFL

**Supplementary Table 5. Model performance with and without the three acute serum biomarkers combined**

Supplementary Table 6. Coefficients for models with and without biomarkers

**Supplementary Table 7. Worst-case sensitivity analysis for missing data age - model performance with and without GFAP**

**Supplementary Table 8. Worst-case sensitivity analysis for missing data age - model performance with and without NFL**

**Supplementary Table 9. Worst-case sensitivity analysis for missing data age - model performance with and without S100B**

**Supplementary Table 10. Worst-case sensitivity analysis for missing data age - model performance with and without three serum biomarkers combined**

**Supplementary Table 11. Best-case sensitivity analysis for missing data age - model performance with and without GFAP**

**Supplementary Table 12. Best-case sensitivity analysis for missing data age - model performance with and without NFL**

**Supplementary Table 13. Best-case sensitivity analysis for missing data age – model performance with and without S100B**

**Supplementary Table 14. Best-case sensitivity analysis for missing data age - model performance with and without three serum biomarkers combined**

**Supplementary Table 15. Sensitivity analysis of NFL timing - model performance with and without 2-3 week NFL**.

Supplementary Table 16. Characteristics of controls in the DTI cohort

Supplementary Table 17. Association of traumatic MRI abnormalities with outcome in univariable logistic regression analyses

Supplementary Table 18. Coefficients for models with and without DTI

**Supplementary Table 19. Sensitivity analysis using DTI data not adjusted for age - model performance with and without DTI**

**Supplementary Table 20. Worst-case sensitivity analysis for missing data age - model performance with and without DTI**

**Supplementary 21. Best-case sensitivity analysis for missing data age - model performance with and without DTI**

Supplementary Table 22. Sample timing and concentration of serum biomarkers

Supplementary Table 23. Using biomarkers to identify patients for DTI, using a range of minimum sensitivities

**Supplementary Table 24. Sensitivity analysis using only patients in the overlap cohort (n = 108) – model performance with and without GFAP**

**Supplementary Table 25. Sensitivity analysis using only patients in the overlap cohort (n = 108) – model performance with and without NFL**

**Supplementary Table 26. Sensitivity analysis using only patients in the overlap cohort (n = 108) – model performance with and without S100B**

**Supplementary Table 27. Sensitivity analysis using only patients in the overlap cohort (n = 108) – model performance with and without all three biomarkers combined**

**Supplementary Table 28. Sensitivity analysis using only patients in the overlap cohort (n = 108) – model performance with and without DTI**

Supplementary Figure 1. Flowchart of patient inclusion

Supplementary Figure 2. Age distribution of the patients with mTBI and healthy volunteers for the participants who were included in the DTI analysis

**Supplementary Figure 3. Association of serum neurofilament light (NFL) with diffusion metrics**

Supplementary Figure 4. Prognostic value of different white matter tracts

Supplementary Figure 5. Current and proposed care pathway based on findings of this study

Supplementary Checklist – TRIPOD Checklist: Prediction Model Development and Validation

**Supplementary Methods 1. Collection and central imputation of the GOSE variable at 3 months**

GOSE was based on the variable Subject.GOSE3monthEndpointDerived available in the database CENTER Core 3·0, accessible via neurobot (https://center-tbi.incf.org). This variable is defined as follows:

“If death occurred on or before 90 days, GOSE was recorded as 1. If there was an observed GOSE recorded in the range of 76 to 125 days (i.e., in the per protocol window for the 3 months assessment point), that value was used. If there is no per-protocol value available then an imputed 90-day GOSE, if available, was used.

Imputation is based on the composite GOSE recorded at different timepoints 2 weeks and one year. The imputation of GOSE uses a multi-state model. The model does not include any baseline covariates. Since the imputation uses GOSE information across timepoints, the imputed 3 month GOSE does not necessarily correspond to outcomes that are observed at 3 months”

The imputation method is based on the following publication: Kunzmann et. al, J Neurotrauma 2021;38(40), doi: https://doi.org/10.1089/neu.2019.6858.

**Supplementary Methods 2. Explanation of the threshold used to choose the most prognostically significant DTI tracts**

Lasso regression is a variable reduction technique (Regression Shrinkage and Selection via the Lasso, Tibshirani, 1996, 10.1111/j.2517-6161.1996.tb02080.x). In lasso regression a penalty factor called lambda is used to shrink the coefficients for all variables in the model (here for all white matter tracts). The higher lambda, the smaller the model coefficients.

As explained in the methods section the magnitude of lambda was chosen through 10x10 cross-validation identifying which size lambda would minimize the mean prediction error.

After shrinkage, the model coefficients for some white matter tracts will be reduced to zero which effectively excludes them from the model. So only white matter tracts with a non-zero coefficient would be retained in the model, considered “most prognostically significant” and be displayed in Table 3.

We conducted our analysis 2000 times (200 bootstrap samples x 10 multiply imputed datasets) and pooled the results. We included in Table 3 any white matter tract that was retained in the lasso model in at least 1 of the 2000 runs of the analysis, and also report in how many of the 2000 analysis runs the tract was selected.

**Supplementary Table 1. Comparison of patients with and without missing data.** ER = discharged from the emergency room, Admission = admitted to a standard ward, ICU = admitted to the intensive care unit, AIS = Abbreviated Injury Severity score, PTA = post-traumatic amnesia, GAD = Generalized Anxiety Disorder assessment, PHQ9 = Patient Health Questionnaire 9, RPQ = Rivermead Post-concussion symptoms Questionnaire, PCL-5 = PTSD Checklist for DSM-5

|  | Biomarker cohort - complete data (N=281) | Biomarker cohort - missing data (N=744) | DTI cohort - complete data (N=84) | DTI cohort - missing data (N=69) | Overlap cohort - complete data (N=61) | Overlap cohort - missing data (N=47) |
| --- | --- | --- | --- | --- | --- | --- |
| **Age (years)** |  |  |  |  |  |  |
| Median (Min-Max) | 47 (18 - 87) | 49 (18 - 93) | 47 (20 - 68) | 41 (20 - 70) | 44 (20 - 68) | 37 (20 - 69) |
| **Sex** |  |  |  |  |  |  |
| female | 122 (43 %) | 247 (33 %) | 27 (32 %) | 18 (26 %) | 19 (31 %) | 11 (23 %) |
| male | 159 (57 %) | 497 (67 %) | 57 (68 %) | 51 (74 %) | 42 (69 %) | 36 (77 %) |
| **Education** |  |  |  |  |  |  |
| below high school diploma | 46 (16 %) | 93 (12 %) | 2 (2 %) | 3 (4 %) | 2 (3 %) | 2 (4 %) |
| high school diploma | 83 (30 %) | 243 (33 %) | 21 (25 %) | 28 (41 %) | 17 (28 %) | 16 (34 %) |
| higher diploma | 53 (19 %) | 160 (22 %) | 16 (19 %) | 11 (16 %) | 11 (18 %) | 7 (15 %) |
| university degree | 99 (35 %) | 178 (24 %) | 45 (54 %) | 22 (32 %) | 31 (51 %) | 19 (40 %) |
| missing | 0 (0%) | 70 (9·4%) | 0 (0%) | 5 (7·2%) | 0 (0%) | 3 (6·4%) |
| **Pre-injury health (ASA)** |  |  |  |  |  |  |
| healthy | 158 (56 %) | 438 (59 %) | 53 (63 %) | 43 (62 %) | 40 (66 %) | 33 (70 %) |
| mild disease | 98 (35 %) | 213 (29 %) | 25 (30 %) | 21 (30 %) | 19 (31 %) | 11 (23 %) |
| severe or life-threatening disease | 25 (9 %) | 83 (11 %) | 6 (7 %) | 5 (7 %) | 2 (3 %) | 3 (6 %) |
| missing | 0 (0%) | 10 (1·3%) | 0 (0%) | 0 (0%) | 0 (0%) | 0 (0%) |
| **Prior mental health problems** |  |  |  |  |  |  |
| absent | 245 (87 %) | 622 (84 %) | 74 (88 %) | 56 (81 %) | 52 (85 %) | 38 (81 %) |
| present | 36 (13 %) | 114 (15 %) | 10 (12 %) | 13 (19 %) | 9 (15 %) | 9 (19 %) |
| missing | 0 (0%) | 8 (1·1%) | 0 (0%) | 0 (0%) | 0 (0%) | 0 (0%) |
| **Cause** |  |  |  |  |  |  |
| fall and other | 172 (61 %) | 413 (56 %) | 38 (45 %) | 29 (42 %) | 28 (46 %) | 18 (38 %) |
| traffic | 93 (33 %) | 262 (35 %) | 44 (52 %) | 28 (41 %) | 33 (54 %) | 22 (47 %) |
| violence | 16 (6 %) | 62 (8 %) | 2 (2 %) | 9 (13 %) | 0 (0 %) | 5 (11 %) |
| missing | 0 (0%) | 7 (0·9%) | 0 (0%) | 3 (4·3%) | 0 (0%) | 2 (4·3%) |
| **Glasgow Coma Score** |  |  |  |  |  |  |
| 13 | 1 (0 %) | 12 (2 %) | 0 (0 %) | 1 (1 %) | 0 (0 %) | 1 (2 %) |
| 14 | 15 (5 %) | 113 (15 %) | 10 (12 %) | 16 (23 %) | 7 (11 %) | 10 (21 %) |
| 15 | 265 (94 %) | 619 (83 %) | 74 (88 %) | 52 (75 %) | 54 (89 %) | 36 (77 %) |
| **Injury severity score** |  |  |  |  |  |  |
| median (min-max) | 5·0 (1 - 32) | 9·0 (1 - 59) | 5·0 (1 - 32) | 8·0 (1 - 41) | 5·0 (1 - 32) | 8·0 (1 - 41) |
| missing | 0 (0%) | 2 (0·3%) | 0 (0%) | 0 (0%) | 0 (0%) | 0 (0%) |
| **Care pathway** |  |  |  |  |  |  |
| ER | 254 (90 %) | 276 (37 %) | 49 (58 %) | 32 (46 %) | 36 (59 %) | 22 (47 %) |
| Admission | 27 (10 %) | 382 (51 %) | 35 (42 %) | 31 (45 %) | 25 (41 %) | 21 (45 %) |
| ICU | 0 (0 %) | 86 (12 %) | 0 (0 %) | 6 (9 %) | 0 (0 %) | 4 (9 %) |
| **Alcohol intoxication** |  |  |  |  |  |  |
| absent | 255 (91 %) | 524 (70 %) | 75 (89 %) | 41 (59 %) | 55 (90 %) | 30 (64 %) |
| present | 26 (9 %) | 111 (15 %) | 9 (11 %) | 16 (23 %) | 6 (10 %) | 8 (17 %) |
| missing | 0 (0%) | 109 (14·7%) | 0 (0%) | 12 (17·4%) | 0 (0%) | 9 (19·1%) |
| **PTA >1h** |  |  |  |  |  |  |
| absent | 255 (91 %) | 539 (72 %) | 71 (85 %) | 42 (61 %) | 52 (85 %) | 25 (53 %) |
| present | 26 (9 %) | 117 (16 %) | 13 (15 %) | 16 (23 %) | 9 (15 %) | 13 (28 %) |
| missing | 0 (0%) | 88 (11·8%) | 0 (0%) | 11 (15·9%) | 0 (0%) | 9 (19·1%) |
| **Time to biomarker (hours)** |  |  |  |  |  |  |
| median (min-max) | 4·8 (0·5 - 23·3) | 11 (0·5 - 24) | 14 (2·1 - 679·4) | 13 (2 - 5425·7) | 8·8 (2·1 - 23·3) | 9·6 (2 - 24) |
| missing | 0 (0%) | 0 (0%) | 4 (4·8%) | 11 (15·9%) | 0 (0%) | 0 (0%) |
| **Time to MRI (days)** |  |  |  |  |  |  |
| 0-3 | 23 (8 %) | 15 (2 %) | 30 (36 %) | 20 (29 %) | 23 (38 %) | 15 (32 %) |
| 4-14 | 13 (5 %) | 7 (1 %) | 17 (20 %) | 18 (26 %) | 13 (21 %) | 7 (15 %) |
| 15-31 | 25 (9 %) | 25 (3 %) | 37 (44 %) | 31 (45 %) | 25 (41 %) | 25 (53 %) |
| missing | 220 (78·3%) | 697 (93·7%) | 0 (0%) | 0 (0%) | 0 (0%) | 0 (0%) |
| **Acute concussion complaints score (RPQ)** |  |  |  |  |  |  |
| median (min-max) | 7·0 (0 - 41) | 6·0 (0 - 46) | 12 (0 - 44) | 9·0 (0 - 37) | 12 (0 - 41) | 9·0 (0 - 37) |
| missing | 0 (0%) | 180 (24·2%) | 0 (0%) | 16 (23·2%) | 0 (0%) | 14 (29·8%) |
| **Moderate or severe headache** |  |  |  |  |  |  |
| absent-mild | 208 (74 %) | 422 (57 %) | 59 (70 %) | 35 (51 %) | 43 (70 %) | 23 (49 %) |
| moderate-severe | 73 (26 %) | 145 (19 %) | 25 (30 %) | 18 (26 %) | 18 (30 %) | 10 (21 %) |
| missing | 0 (0%) | 177 (23·8%) | 0 (0%) | 16 (23·2%) | 0 (0%) | 14 (29·8%) |
| **Mild or worse difficulty concentrating** |  |  |  |  |  |  |
| absent | 211 (75 %) | 442 (59 %) | 50 (60 %) | 38 (55 %) | 33 (54 %) | 23 (49 %) |
| present | 70 (25 %) | 119 (16 %) | 34 (40 %) | 13 (19 %) | 28 (46 %) | 8 (17 %) |
| missing | 0 (0%) | 183 (24·6%) | 0 (0%) | 18 (26·1%) | 0 (0%) | 16 (34·0%) |
| **Mild or worse photophobia** |  |  |  |  |  |  |
| absent | 235 (84 %) | 487 (65 %) | 60 (71 %) | 39 (57 %) | 43 (70 %) | 22 (47 %) |
| present | 46 (16 %) | 76 (10 %) | 24 (29 %) | 13 (19 %) | 18 (30 %) | 10 (21 %) |
| missing | 0 (0%) | 181 (24·3%) | 0 (0%) | 17 (24·6%) | 0 (0%) | 15 (31·9%) |
| **2wk anxiety score (GAD)** |  |  |  |  |  |  |
| median (min-max) | 2·0 (0 - 21) | 3·0 (0 - 21) | 2·0 (0 - 14) | 3·0 (0 - 16) | 2·0 (0 - 14) | 3·0 (0 - 14) |
| missing | 0 (0%) | 642 (86·3%) | 0 (0%) | 41 (59·4%) | 0 (0%) | 22 (46·8%) |
| **2wk depression score (PHQ9)** |  |  |  |  |  |  |
| median (min-max) | 5·0 (0 - 24) | 6·0 (0 - 27) | 6·0 (0 - 19) | 7·0 (0 - 27) | 7·0 (0 - 19) | 7·5 (0 - 17) |
| missing | 0 (0%) | 645 (86·7%) | 0 (0%) | 40 (58·0%) | 0 (0%) | 23 (48·9%) |
| **2wk concussion complaints score (RPQ)** |  |  |  |  |  |  |
| median (min-max) | 6·0 (0 - 51) | 7·0 (0 - 53) | 9·0 (0 - 44) | 8·0 (0 - 43) | 10 (0 - 44) | 9·0 (0 - 43) |
| missing | 0 (0%) | 639 (85·9%) | 0 (0%) | 38 (55·1%) | 0 (0%) | 21 (44·7%) |
| **2wk post-traumatic stress score (PCL-5)** |  |  |  |  |  |  |
| median (min-max) | 8·0 (0 - 66) | 11 (0 - 72) | 9·5 (0 - 51) | 9·0 (1 - 42) | 9·0 (0 - 38) | 9·0 (2 - 42) |
| missing | 0 (0%) | 649 (87·2%) | 0 (0%) | 41 (59·4%) | 0 (0%) | 23 (48·9%) |
| **Recovery** |  |  |  |  |  |  |
| complete | 167 (59 %) | 293 (39 %) | 41 (49 %) | 38 (55 %) | 34 (56 %) | 26 (55 %) |
| incomplete | 114 (41 %) | 271 (36 %) | 43 (51 %) | 27 (39 %) | 27 (44 %) | 20 (43 %) |
| missing | 0 (0%) | 180 (24·2%) | 0 (0%) | 4 (5·8%) | 0 (0%) | 1 (2·1%) |

Supplementary Table 2. Characteristics of included patients**.** ER = discharged from the emergency room, Admission = admitted to a standard ward, ICU = admitted to the intensive care unit, AIS = Abbreviated Injury Severity score, PTA = post-traumatic amnesia, GAD = Generalized Anxiety Disorder assessment, PHQ9 = Patient Health Questionnaire 9, RPQ = Rivermead Post-concussion symptoms Questionnaire, PCL-5 = PTSD Checklist for DSM-5

|  | All adults with mTBI and normal CT (N=1408) | Biomarker cohort (N=1025) | DTI cohort (N=153) | Overlap cohort (N=108) |
| --- | --- | --- | --- | --- |
| **Age (years)** |  |  |  |  |
| Median (Min-Max) | 49 (18 - 94) | 48 (18 - 93) | 44 (20 - 70) | 41 (20 - 69) |
| **Sex** |  |  |  |  |
| female | 531 (38 %) | 369 (36 %) | 45 (29 %) | 30 (28 %) |
| male | 877 (62 %) | 656 (64 %) | 108 (71 %) | 78 (72 %) |
| **Education** |  |  |  |  |
| below high school diploma | 184 (13 %) | 139 (14 %) | 5 (3 %) | 4 (4 %) |
| high school diploma | 442 (31 %) | 326 (32 %) | 49 (32 %) | 33 (31 %) |
| higher diploma | 304 (22 %) | 213 (21 %) | 27 (18 %) | 18 (17 %) |
| university degree | 374 (27 %) | 277 (27 %) | 67 (44 %) | 50 (46 %) |
| missing | 104 (7·4%) | 70 (6·8%) | 5 (3·3%) | 3 (2·8%) |
| **Pre-injury health (ASA)** |  |  |  |  |
| healthy | 794 (56 %) | 596 (58 %) | 96 (63 %) | 73 (68 %) |
| mild disease | 448 (32 %) | 311 (30 %) | 46 (30 %) | 30 (28 %) |
| severe or life-threatening disease | 154 (11 %) | 108 (11 %) | 11 (7 %) | 5 (5 %) |
| missing | 12 (0·9%) | 10 (1·0%) | 0 (0%) | 0 (0%) |
| **Prior mental health problems** |  |  |  |  |
| absent | 1198 (85 %) | 867 (85 %) | 130 (85 %) | 90 (83 %) |
| present | 201 (14 %) | 150 (15 %) | 23 (15 %) | 18 (17 %) |
| missing | 9 (0·6%) | 8 (0·8%) | 0 (0%) | 0 (0%) |
| **Cause** |  |  |  |  |
| fall and other | 794 (56 %) | 585 (57 %) | 67 (44 %) | 46 (43 %) |
| traffic | 501 (36 %) | 355 (35 %) | 72 (47 %) | 55 (51 %) |
| violence | 103 (7 %) | 78 (8 %) | 11 (7 %) | 5 (5 %) |
| missing | 10 (0·7%) | 7 (0·7%) | 3 (2·0%) | 2 (1·9%) |
| **Glasgow Coma Score** |  |  |  |  |
| 13 | 19 (1 %) | 13 (1 %) | 1 (1 %) | 1 (1 %) |
| 14 | 180 (13 %) | 128 (12 %) | 26 (17 %) | 17 (16 %) |
| 15 | 1209 (86 %) | 884 (86 %) | 126 (82 %) | 90 (83 %) |
| **Injury severity score** |  |  |  |  |
| median (min-max) | 8·0 (1 - 59) | 8·0 (1 - 59) | 6·0 (1 - 41) | 6·0 (1 - 41) |
| missing | 4 (0·3%) | 2 (0·2%) | 0 (0%) | 0 (0%) |
| **Care pathway** |  |  |  |  |
| ER | 681 (48 %) | 530 (52 %) | 81 (53 %) | 58 (54 %) |
| Admission | 615 (44 %) | 409 (40 %) | 66 (43 %) | 46 (43 %) |
| ICU | 112 (8 %) | 86 (8 %) | 6 (4 %) | 4 (4 %) |
| **Alcohol intoxication** |  |  |  |  |
| absent | 1062 (75 %) | 779 (76 %) | 116 (76 %) | 85 (79 %) |
| present | 204 (14 %) | 137 (13 %) | 25 (16 %) | 14 (13 %) |
| missing | 142 (10·1%) | 109 (10·6%) | 12 (7·8%) | 9 (8·3%) |
| **PTA >1h** |  |  |  |  |
| absent | 1108 (79 %) | 794 (77 %) | 113 (74 %) | 77 (71 %) |
| present | 180 (13 %) | 143 (14 %) | 29 (19 %) | 22 (20 %) |
| missing | 120 (8·5%) | 88 (8·6%) | 11 (7·2%) | 9 (8·3%) |
| **Time to biomarker (hours)** |  |  |  |  |
| median (min-max) | 11 (0·5 - 11872·4) | 8·8 (0·5 - 24) | 14 (2 - 5425·7) | 9·5 (2 - 24) |
| missing | 246 (17·5%) | 0 (0%) | 15 (9·8%) | 0 (0%) |
| **Time to MRI (days)** |  |  |  |  |
| 0-3 | 50 (4 %) | 38 (4 %) | 50 (33 %) | 38 (35 %) |
| 4-14 | 35 (2 %) | 20 (2 %) | 35 (23 %) | 20 (19 %) |
| 15-31 | 68 (5 %) | 50 (5 %) | 68 (44 %) | 50 (46 %) |
| missing | 1255 (89·1%) | 917 (89·5%) | 0 (0%) | 0 (0%) |
| **Acute concussion complaints score (RPQ)** |  |  |  |  |
| median (min-max) | 7·0 (0 - 49) | 7·0 (0 - 46) | 10 (0 - 44) | 11 (0 - 41) |
| missing | 232 (16·5%) | 180 (17·6%) | 16 (10·5%) | 14 (13·0%) |
| **Moderate or severe headache** |  |  |  |  |
| absent-mild | 854 (61 %) | 630 (61 %) | 94 (61 %) | 66 (61 %) |
| moderate-severe | 326 (23 %) | 218 (21 %) | 43 (28 %) | 28 (26 %) |
| missing | 228 (16·2%) | 177 (17·3%) | 16 (10·5%) | 14 (13·0%) |
| **Mild or worse difficulty concentrating** |  |  |  |  |
| absent | 905 (64 %) | 653 (64 %) | 88 (58 %) | 56 (52 %) |
| present | 268 (19 %) | 189 (18 %) | 47 (31 %) | 36 (33 %) |
| missing | 235 (16·7%) | 183 (17·9%) | 18 (11·8%) | 16 (14·8%) |
| **Mild or worse photophobia** |  |  |  |  |
| absent | 1000 (71 %) | 722 (70 %) | 99 (65 %) | 65 (60 %) |
| present | 175 (12 %) | 122 (12 %) | 37 (24 %) | 28 (26 %) |
| missing | 233 (16·5%) | 181 (17·7%) | 17 (11·1%) | 15 (13·9%) |
| **2wk anxiety score (GAD)** |  |  |  |  |
| median (min-max) | 2·0 (0 - 21) | 2·0 (0 - 21) | 3·0 (0 - 16) | 3·0 (0 - 14) |
| missing | 934 (66·3%) | 642 (62·6%) | 41 (26·8%) | 22 (20·4%) |
| **2wk depression score (PHQ9)** |  |  |  |  |
| median (min-max) | 5·0 (0 - 27) | 5·0 (0 - 27) | 6·0 (0 - 27) | 7·0 (0 - 19) |
| missing | 935 (66·4%) | 645 (62·9%) | 40 (26·1%) | 23 (21·3%) |
| **2wk concussion complaints score (RPQ)** |  |  |  |  |
| median (min-max) | 6·0 (0 - 54) | 6·0 (0 - 53) | 8·0 (0 - 44) | 10 (0 - 44) |
| missing | 926 (65·8%) | 639 (62·3%) | 38 (24·8%) | 21 (19·4%) |
| **2wk post-traumatic stress score (PCL-5)** |  |  |  |  |
| median (min-max) | 8·0 (0 - 72) | 9·0 (0 - 72) | 9·0 (0 - 51) | 9·0 (0 - 42) |
| missing | 941 (66·8%) | 649 (63·3%) | 41 (26·8%) | 23 (21·3%) |
| **Recovery** |  |  |  |  |
| complete | 609 (43 %) | 460 (45 %) | 79 (52 %) | 60 (56 %) |
| incomplete | 544 (39 %) | 385 (38 %) | 70 (46 %) | 47 (44 %) |
| missing | 255 (18·1%) | 180 (17·6%) | 4 (2·6%) | 1 (0·9%) |

Supplementary Table 3. Model performance with and without early GFAP

| Metric | GFAP only | UPFRONT-ED | | HeadSMART | | CENTER-ED | |
| --- | --- | --- | --- | --- | --- | --- | --- |
|  |  | w/o GFAP | with GFAP | w/o GFAP | with GFAP | w/o GFAP | with GFAP |
| Area under the curve | 0·56 (0·52-0·59) | 0·57 (0·53-0·61) | 0·57 (0·52-0·61) | 0·58 (0·54-0·62) | 0·59 (0·54-0·63) | 0·69 (0·65-0·72) | 0·69 (0·65-0·73) |
| Variation explained (%) | 0 (-1-1) | 5 (1-8) | 5 (1-8) | 4 (1-8) | 4 (1-8) | 17 (11-22) | 17 (11-22) |
| Sensitivity | 0·00 (-0·09-0·10) | 0·28 (0·16-0·40) | 0·28 (0·16-0·40) | 0·33 (0·20-0·45) | 0·33 (0·20-0·45) | 0·48 (0·40-0·56) | 0·48 (0·41-0·56) |
| Specificity | 0·99 (0·93-1·05) | 0·82 (0·73-0·90) | 0·81 (0·73-0·90) | 0·81 (0·73-0·88) | 0·80 (0·73-0·88) | 0·77 (0·72-0·83) | 0·78 (0·72-0·83) |
| Positive predictive value | NaN (NaN-NaN) | 0·56 (0·50-0·62) | 0·56 (0·50-0·62) | 0·58 (0·51-0·65) | 0·58 (0·51-0·65) | 0·64 (0·58-0·69) | 0·64 (0·59-0·69) |
| Negative predictive value | NaN (NaN-NaN) | 0·58 (0·54-0·61) | 0·58 (0·54-0·62) | 0·59 (0·56-0·63) | 0·59 (0·56-0·63) | 0·65 (0·61-0·68) | 0·65 (0·61-0·68) |
| Likelihood ratio test p-value |  |  | 0·578 |  | 0·396 |  | 0·297 |

Supplementary Table 4. Model performance with and without early NFL

| Metric | NFL only | UPFRONT-ED | | HeadSMART | | CENTER-ED | |
| --- | --- | --- | --- | --- | --- | --- | --- |
|  |  | w/o NFL | with NFL | w/o NFL | with NFL | w/o NFL | with NFL |
| Area under the curve | 0·54 (0·50-0·58) | 0·57 (0·53-0·61) | 0·58 (0·53-0·62) | 0·58 (0·54-0·62) | 0·59 (0·55-0·63) | 0·69 (0·65-0·72) | 0·69 (0·65-0·72) |
| Variation explained (%) | 1 (-1-2) | 5 (1-8) | 5 (1-9) | 4 (1-8) | 5 (1-8) | 17 (11-22) | 17 (11-22) |
| Sensitivity | 0·02 (-0·07-0·12) | 0·28 (0·16-0·40) | 0·29 (0·18-0·40) | 0·33 (0·20-0·45) | 0·33 (0·20-0·45) | 0·48 (0·40-0·56) | 0·48 (0·40-0·56) |
| Specificity | 0·99 (0·94-1·03) | 0·82 (0·73-0·90) | 0·81 (0·73-0·90) | 0·81 (0·73-0·88) | 0·80 (0·73-0·88) | 0·77 (0·72-0·83) | 0·77 (0·72-0·83) |
| Positive predictive value | 0·72 (0·54-0·90) | 0·56 (0·50-0·62) | 0·57 (0·50-0·63) | 0·58 (0·51-0·65) | 0·57 (0·50-0·65) | 0·64 (0·58-0·69) | 0·64 (0·58-0·69) |
| Negative predictive value | 0·56 (0·53-0·58) | 0·58 (0·54-0·61) | 0·58 (0·55-0·62) | 0·59 (0·56-0·63) | 0·59 (0·56-0·63) | 0·65 (0·61-0·68) | 0·65 (0·61-0·68) |
| Likelihood ratio test p-value |  |  | 0·065 |  | 0·113 |  | 0·390 |

**Supplementary Table 5. Model performance with and without the three early serum biomarkers combined**

| Metric | With GFAP, NFL & S100B only | UPFRONT-ED | | HeadSMART | | CENTER-ED | |
| --- | --- | --- | --- | --- | --- | --- | --- |
|  |  | w/o GFAP, NFL & S100B | with GFAP, NFL & S100B | w/o GFAP, NFL & S100B | with GFAP, NFL & S100B | w/o GFAP, NFL & S100B | with GFAP, NFL & S100B |
| Area under the curve | 0·57 (0·53-0·61) | 0·57 (0·53-0·61) | 0·60 (0·56-0·64) | 0·58 (0·54-0·62) | 0·62 (0·58-0·66) | 0·69 (0·65-0·72) | 0·69 (0·66-0·73) |
| Variation explained (%) | 3 (0-6) | 5 (1-8) | 7 (3-12) | 4 (1-8) | 7 (3-11) | 17 (11-22) | 18 (12-24) |
| Sensitivity | 0·15 (0·03-0·27) | 0·28 (0·16-0·40) | 0·34 (0·23-0·44) | 0·33 (0·20-0·45) | 0·36 (0·25-0·48) | 0·48 (0·40-0·56) | 0·48 (0·40-0·56) |
| Specificity | 0·92 (0·86-0·98) | 0·82 (0·73-0·90) | 0·80 (0·73-0·87) | 0·81 (0·73-0·88) | 0·79 (0·73-0·86) | 0·77 (0·72-0·83) | 0·77 (0·72-0·83) |
| Positive predictive value | 0·62 (0·54-0·70) | 0·56 (0·50-0·62) | 0·58 (0·53-0·63) | 0·58 (0·51-0·65) | 0·59 (0·53-0·65) | 0·64 (0·58-0·69) | 0·64 (0·59-0·69) |
| Negative predictive value | 0·57 (0·54-0·60) | 0·58 (0·54-0·61) | 0·59 (0·56-0·63) | 0·59 (0·56-0·63) | 0·60 (0·57-0·64) | 0·65 (0·61-0·68) | 0·65 (0·61-0·68) |
| Likelihood ratio test p-value |  |  | 0·001 |  | <0·001 |  | 0·063 |

Supplementary Table 6. Coefficients for models with and without early biomarkers. Model coefficients were pooled across the 10 imputed datasets. LL and UL = lower level and upper level of the 95% confidence interval. Sig = statistical significance recorded as either significant (signif) or not significant (ns).

| **Model** | **Variable** | **Pooled beta** | **LL** | **UL** | **Sig** |
| --- | --- | --- | --- | --- | --- |
| GFAP only | (Intercept) | -0·217 | -0·359 | -0·076 | signif |
|  | GFAP | 0·008 | -0·020 | 0·037 | ns |
| NFL only | (Intercept) | -0·251 | -0·405 | -0·098 | signif |
|  | NFL | 0·003 | -0·002 | 0·008 | ns |
| S100B only | (Intercept) | -0·422 | -0·600 | -0·245 | signif |
|  | S100B | 1·594 | 0·671 | 2·517 | signif |
| UPFRONT-ED | (Intercept) | 2·194 | 0·348 | 4·040 | signif |
|  | Sex - male | -0·195 | -0·521 | 0·131 | ns |
|  | Education - GCSE/AS/A levels equivalent | -1·029 | -2·343 | 0·284 | ns |
|  | Education - diploma | -0·872 | -2·259 | 0·516 | ns |
|  | Education - university degree | -1·350 | -2·703 | 0·002 | ns |
|  | Age | -0·014 | -0·032 | 0·005 | ns |
|  | Mental health problems - present | 0·658 | 0·269 | 1·047 | signif |
|  | Alcohol intoxication - present | -0·266 | -0·715 | 0·183 | ns |
|  | GCSScoreBaselineDerived14 | -1·014 | -2·420 | 0·391 | ns |
|  | GCS - 15 | -1·347 | -2·715 | 0·020 | ns |
|  | PTA over 1h - present | -0·069 | -0·493 | 0·354 | ns |
|  | Education - GCSE/AS/A levels equivalent:Age | 0·013 | -0·009 | 0·035 | ns |
|  | Education - diploma:Age | 0·008 | -0·016 | 0·032 | ns |
|  | Education - university degree:Age | 0·021 | -0·004 | 0·045 | ns |
| HeadSMART | (Intercept) | -0·556 | -0·973 | -0·139 | signif |
|  | Age | 0·002 | -0·005 | 0·010 | ns |
|  | Mental health problems - present | 0·620 | 0·237 | 1·002 | signif |
|  | moderate-severe headache | -0·077 | -0·467 | 0·314 | ns |
|  | poor concentration | 0·243 | -0·093 | 0·579 | ns |
|  | light sensitivity | 0·635 | 0·152 | 1·119 | signif |
| CENTER-ED | (Intercept) | -1·226 | -2·904 | 0·453 | ns |
|  | Age | 0·003 | -0·007 | 0·013 | ns |
|  | ISS | 0·060 | 0·039 | 0·082 | signif |
|  | GCSScoreBaselineDerived14 | -0·275 | -1·759 | 1·209 | ns |
|  | GCS - 15 | -0·283 | -1·726 | 1·159 | ns |
|  | Sex - male | -0·305 | -0·673 | 0·062 | ns |
|  | Mental health problems - present | 0·466 | 0·041 | 0·892 | signif |
|  | ASA - mild disease | 0·267 | -0·084 | 0·618 | ns |
|  | ASA - severe or life-threatening disease | 0·471 | -0·099 | 1·040 | ns |
|  | Cause of injury - traffic | 0·701 | 0·354 | 1·049 | signif |
|  | Cause of injury - violence | 0·049 | -0·698 | 0·796 | ns |
|  | acute RPQ score (concussion) | 0·037 | 0·019 | 0·054 | signif |
| UPFRONT-ED with GFAP | (Intercept) | 2·165 | 0·320 | 4·010 | signif |
|  | Sex - male | -0·197 | -0·523 | 0·130 | ns |
|  | Education - GCSE/AS/A levels equivalent | -1·034 | -2·347 | 0·280 | ns |
|  | Education - diploma | -0·871 | -2·257 | 0·516 | ns |
|  | Education - university degree | -1·344 | -2·696 | 0·007 | ns |
|  | Age | -0·014 | -0·032 | 0·005 | ns |
|  | Mental health problems - present | 0·664 | 0·275 | 1·052 | signif |
|  | Alcohol intoxication - present | -0·264 | -0·713 | 0·186 | ns |
|  | GCSScoreBaselineDerived14 | -1·005 | -2·411 | 0·401 | ns |
|  | GCS - 15 | -1·331 | -2·699 | 0·038 | ns |
|  | PTA over 1h - present | -0·074 | -0·496 | 0·349 | ns |
|  | GFAP | 0·008 | -0·020 | 0·036 | ns |
|  | Education - GCSE/AS/A levels equivalent:Age | 0·013 | -0·009 | 0·035 | ns |
|  | Education - diploma:Age | 0·008 | -0·016 | 0·032 | ns |
|  | Education - university degree:Age | 0·020 | -0·004 | 0·045 | ns |
| HeadSMART with GFAP | (Intercept) | -0·580 | -0·998 | -0·162 | signif |
|  | Age | 0·003 | -0·005 | 0·010 | ns |
|  | Mental health problems - present | 0·628 | 0·245 | 1·011 | signif |
|  | moderate-severe headache | -0·075 | -0·466 | 0·317 | ns |
|  | poor concentration | 0·241 | -0·095 | 0·577 | ns |
|  | light sensitivity | 0·638 | 0·155 | 1·122 | signif |
|  | GFAP | 0·012 | -0·017 | 0·042 | ns |
| CENTER-ED with GFAP | (Intercept) | -1·193 | -2·873 | 0·487 | ns |
|  | Age | 0·003 | -0·007 | 0·013 | ns |
|  | ISS | 0·062 | 0·040 | 0·085 | signif |
|  | GCSScoreBaselineDerived14 | -0·284 | -1·772 | 1·205 | ns |
|  | GCS - 15 | -0·304 | -1·750 | 1·141 | ns |
|  | Sex - male | -0·307 | -0·674 | 0·060 | ns |
|  | Mental health problems - present | 0·456 | 0·032 | 0·881 | signif |
|  | ASA - mild disease | 0·261 | -0·091 | 0·613 | ns |
|  | ASA - severe or life-threatening disease | 0·468 | -0·102 | 1·039 | ns |
|  | Cause of injury - traffic | 0·713 | 0·362 | 1·063 | signif |
|  | Cause of injury - violence | 0·035 | -0·711 | 0·782 | ns |
|  | acute RPQ score (concussion) | 0·036 | 0·018 | 0·054 | signif |
|  | GFAP | -0·020 | -0·063 | 0·023 | ns |
|  |  |  |  |  |  |
| UPFRONT-ED with NFL | (Intercept) | 2·223 | 0·373 | 4·073 | signif |
|  | Sex - male | -0·207 | -0·536 | 0·123 | ns |
|  | Education - GCSE/AS/A levels equivalent | -1·067 | -2·381 | 0·247 | ns |
|  | Education - diploma | -0·886 | -2·275 | 0·502 | ns |
|  | Education - university degree | -1·376 | -2·727 | -0·025 | signif |
|  | Age | -0·015 | -0·034 | 0·003 | ns |
|  | Mental health problems - present | 0·659 | 0·270 | 1·049 | signif |
|  | Alcohol intoxication - present | -0·273 | -0·723 | 0·178 | ns |
|  | GCSScoreBaselineDerived14 | -1·013 | -2·426 | 0·400 | ns |
|  | GCS - 15 | -1·331 | -2·706 | 0·044 | ns |
|  | PTA over 1h - present | -0·063 | -0·488 | 0·362 | ns |
|  | NFL | 0·003 | -0·003 | 0·009 | ns |
|  | Education - GCSE/AS/A levels equivalent:Age | 0·013 | -0·009 | 0·036 | ns |
|  | Education - diploma:Age | 0·008 | -0·016 | 0·032 | ns |
|  | Education - university degree:Age | 0·021 | -0·003 | 0·046 | ns |
| HeadSMART with NFL | (Intercept) | -0·546 | -0·966 | -0·126 | signif |
|  | Age | 0·001 | -0·007 | 0·009 | ns |
|  | Mental health problems - present | 0·619 | 0·235 | 1·002 | signif |
|  | moderate-severe headache | -0·077 | -0·458 | 0·304 | ns |
|  | poor concentration | 0·253 | -0·083 | 0·589 | ns |
|  | light sensitivity | 0·623 | 0·155 | 1·092 | signif |
|  | NFL | 0·003 | -0·003 | 0·009 | ns |
| CENTER-ED with NFL | (Intercept) | -1·208 | -2·887 | 0·471 | ns |
|  | Age | 0·002 | -0·008 | 0·013 | ns |
|  | ISS | 0·060 | 0·038 | 0·081 | signif |
|  | GCSScoreBaselineDerived14 | -0·280 | -1·766 | 1·206 | ns |
|  | GCS - 15 | -0·288 | -1·732 | 1·155 | ns |
|  | Sex - male | -0·311 | -0·682 | 0·060 | ns |
|  | Mental health problems - present | 0·469 | 0·044 | 0·894 | signif |
|  | ASA - mild disease | 0·262 | -0·088 | 0·612 | ns |
|  | ASA - severe or life-threatening disease | 0·433 | -0·137 | 1·003 | ns |
|  | Cause of injury - traffic | 0·706 | 0·358 | 1·054 | signif |
|  | Cause of injury - violence | 0·054 | -0·692 | 0·801 | ns |
|  | acute RPQ score (concussion) | 0·036 | 0·018 | 0·054 | signif |
|  | NFL | 0·002 | -0·003 | 0·006 | ns |
| UPFRONT-ED with S100B | (Intercept) | 1·955 | 0·078 | 3·832 | signif |
|  | Sex - male | -0·165 | -0·494 | 0·163 | ns |
|  | Education - GCSE/AS/A levels equivalent | -1·147 | -2·459 | 0·166 | ns |
|  | Education - diploma | -0·884 | -2·282 | 0·514 | ns |
|  | Education - university degree | -1·418 | -2·772 | -0·065 | signif |
|  | Age | -0·015 | -0·033 | 0·004 | ns |
|  | Mental health problems - present | 0·680 | 0·289 | 1·071 | signif |
|  | Alcohol intoxication - present | -0·241 | -0·691 | 0·209 | ns |
|  | GCSScoreBaselineDerived14 | -0·976 | -2·397 | 0·445 | ns |
|  | GCS - 15 | -1·278 | -2·660 | 0·103 | ns |
|  | PTA over 1h - present | -0·090 | -0·516 | 0·336 | ns |
|  | S100B | 1·577 | 0·618 | 2·536 | signif |
|  | Education - GCSE/AS/A levels equivalent:Age | 0·015 | -0·007 | 0·037 | ns |
|  | Education - diploma:Age | 0·009 | -0·015 | 0·033 | ns |
|  | Education - university degree:Age | 0·022 | -0·003 | 0·047 | ns |
| HeadSMART with S100B | (Intercept) | -0·801 | -1·237 | -0·364 | signif |
|  | Age | 0·002 | -0·005 | 0·010 | ns |
|  | Mental health problems - present | 0·633 | 0·247 | 1·020 | signif |
|  | moderate-severe headache | -0·044 | -0·442 | 0·354 | ns |
|  | poor concentration | 0·259 | -0·077 | 0·595 | ns |
|  | light sensitivity | 0·627 | 0·140 | 1·114 | signif |
|  | S100B | 1·687 | 0·710 | 2·665 | signif |
| CENTER-ED with S100B | (Intercept) | -1·335 | -3·024 | 0·355 | ns |
|  | Age | 0·003 | -0·007 | 0·013 | ns |
|  | ISS | 0·056 | 0·033 | 0·079 | signif |
|  | GCSScoreBaselineDerived14 | -0·296 | -1·784 | 1·192 | ns |
|  | GCS - 15 | -0·301 | -1·746 | 1·145 | ns |
|  | Sex - male | -0·266 | -0·636 | 0·104 | ns |
|  | Mental health problems - present | 0·470 | 0·044 | 0·896 | signif |
|  | ASA - mild disease | 0·268 | -0·084 | 0·620 | ns |
|  | ASA - severe or life-threatening disease | 0·452 | -0·121 | 1·024 | ns |
|  | Cause of injury - traffic | 0·679 | 0·330 | 1·028 | signif |
|  | Cause of injury - violence | 0·053 | -0·699 | 0·805 | ns |
|  | acute RPQ score (concussion) | 0·038 | 0·020 | 0·056 | signif |
|  | S100B | 0·953 | -0·093 | 1·999 | ns |

**Supplementary Table 7. Worst-case sensitivity analysis for missing data age - model performance with and without GFAP**

| Metric | GFAP only | UPFRONT-ED | | HeadSMART | | CENTER-ED | |
| --- | --- | --- | --- | --- | --- | --- | --- |
|  |  | w/o GFAP | with GFAP | w/o GFAP | with GFAP | w/o GFAP | with GFAP |
| Area under the curve | 0·53 (0·49-0·56) | 0·56 (0·53-0·59) | 0·56 (0·52-0·59) | 0·56 (0·53-0·60) | 0·57 (0·53-0·61) | 0·65 (0·61-0·68) | 0·65 (0·61-0·68) |
| Variation explained (%) | 0 (-1-1) | 4 (1-7) | 4 (1-7) | 3 (1-6) | 3 (1-6) | 11 (7-15) | 11 (7-16) |
| Sensitivity | 1·00 (0·98-1·02) | 0·74 (0·59-0·89) | 0·74 (0·59-0·89) | 0·75 (0·20-1·29) | 0·75 (0·27-1·24) | 0·67 (0·60-0·75) | 0·67 (0·59-0·75) |
| Specificity | 0·00 (-0·02-0·02) | 0·32 (0·12-0·52) | 0·32 (0·12-0·52) | 0·29 (-0·41-0·99) | 0·29 (-0·34-0·92) | 0·51 (0·39-0·62) | 0·50 (0·39-0·62) |
| Positive predictive value | - | 0·57 (0·53-0·61) | 0·57 (0·53-0·60) | - | - | 0·63 (0·59-0·66) | 0·63 (0·59-0·66) |
| Negative predictive value | - | 0·50 (0·45-0·55) | 0·50 (0·45-0·55) | - | - | 0·56 (0·52-0·60) | 0·56 (0·52-0·60) |
| Likelihood ratio test p-value |  |  | 0·948 |  | 0·825 |  | 0·248 |

**Supplementary Table 8. Worst-case sensitivity analysis for missing data age - model performance with and without NFL**

| Metric | NFL only | UPFRONT-ED | | HeadSMART | | CENTER-ED | |
| --- | --- | --- | --- | --- | --- | --- | --- |
|  |  | w/o NFL | with NFL | w/o NFL | with NFL | w/o NFL | with NFL |
| Area under the curve | 0·53 (0·50-0·57) | 0·56 (0·53-0·59) | 0·57 (0·54-0·60) | 0·56 (0·53-0·60) | 0·58 (0·55-0·62) | 0·65 (0·61-0·68) | 0·65 (0·61-0·68) |
| Variation explained (%) | 1 (0-2) | 4 (1-7) | 5 (2-8) | 3 (1-6) | 4 (1-7) | 11 (7-15) | 11 (7-16) |
| Sensitivity | 1·00 (0·91-1·09) | 0·74 (0·59-0·89) | 0·72 (0·58-0·86) | 0·75 (0·20-1·29) | 0·66 (0·36-0·97) | 0·67 (0·60-0·75) | 0·67 (0·59-0·75) |
| Specificity | 0·00 (-0·11-0·11) | 0·32 (0·12-0·52) | 0·35 (0·16-0·54) | 0·29 (-0·41-0·99) | 0·44 (0·04-0·84) | 0·51 (0·39-0·62) | 0·51 (0·40-0·63) |
| Positive predictive value | - | 0·57 (0·53-0·61) | 0·58 (0·54-0·61) | - | - | 0·63 (0·59-0·66) | 0·63 (0·59-0·66) |
| Negative predictive value | - | 0·50 (0·45-0·55) | 0·51 (0·46-0·56) | - | - | 0·56 (0·52-0·60) | 0·56 (0·52-0·60) |
| Likelihood ratio test p-value |  |  | 0·011 |  | 0·012 |  | 0·090 |

**Supplementary Table 9. Worst-case sensitivity analysis for missing data age - model performance with and without S100B**

| Metric | S100B only | UPFRONT-ED | | HeadSMART | | CENTER-ED | |
| --- | --- | --- | --- | --- | --- | --- | --- |
|  |  | w/o S100B | with S100B | w/o S100B | with S100B | w/o S100B | with S100B |
| Area under the curve | 0·56 (0·52-0·59) | 0·56 (0·53-0·59) | 0·58 (0·55-0·62) | 0·56 (0·53-0·60) | 0·60 (0·56-0·63) | 0·65 (0·61-0·68) | 0·65 (0·62-0·69) |
| Variation explained (%) | 2 (0-4) | 4 (1-7) | 6 (2-9) | 3 (1-6) | 5 (2-9) | 11 (7-15) | 12 (7-16) |
| Sensitivity | 1·00 (0·76-1·23) | 0·74 (0·59-0·89) | 0·69 (0·55-0·83) | 0·75 (0·20-1·29) | 0·63 (0·43-0·84) | 0·67 (0·60-0·75) | 0·68 (0·60-0·76) |
| Specificity | 0·00 (-0·28-0·28) | 0·32 (0·12-0·52) | 0·41 (0·22-0·60) | 0·29 (-0·41-0·99) | 0·51 (0·22-0·81) | 0·51 (0·39-0·62) | 0·54 (0·42-0·65) |
| Positive predictive value | - | 0·57 (0·53-0·61) | 0·59 (0·55-0·63) | - | 0·62 (0·57-0·67) | 0·63 (0·59-0·66) | 0·64 (0·61-0·68) |
| Negative predictive value | - | 0·50 (0·45-0·55) | 0·52 (0·47-0·57) | - | 0·53 (0·49-0·58) | 0·56 (0·52-0·60) | 0·58 (0·54-0·62) |
| Likelihood ratio test p-value |  |  | <0·001 |  | <0·001 |  | 0·027 |

**Supplementary Table 10. Worst-case sensitivity analysis for missing data age - model performance with and without three serum biomarkers combined**

| Metric | GFAP, NFL & S100B only | UPFRONT-ED | | HeadSMART | | CENTER-ED | |
| --- | --- | --- | --- | --- | --- | --- | --- |
|  |  | w/o GFAP, NFL & S100B | with GFAP, NFL & S100B | w/o GFAP, NFL & S100B | with GFAP, NFL & S100B | w/o GFAP, NFL & S100B | with GFAP, NFL & S100B |
| Area under the curve | 0·56 (0·52-0·60) | 0·56 (0·53-0·59) | 0·59 (0·56-0·63) | 0·56 (0·53-0·60) | 0·60 (0·57-0·64) | 0·65 (0·61-0·68) | 0·66 (0·63-0·69) |
| Variation explained (%) | 3 (0-5) | 4 (1-7) | 7 (3-10) | 3 (1-6) | 6 (3-10) | 11 (7-15) | 12 (8-17) |
| Sensitivity | 0·98 (0·70-1·25) | 0·74 (0·59-0·89) | 0·68 (0·55-0·81) | 0·75 (0·20-1·29) | 0·64 (0·47-0·81) | 0·67 (0·60-0·75) | 0·68 (0·60-0·76) |
| Specificity | 0·04 (-0·28-0·36) | 0·32 (0·12-0·52) | 0·44 (0·27-0·61) | 0·29 (-0·41-0·99) | 0·50 (0·25-0·75) | 0·51 (0·39-0·62) | 0·54 (0·43-0·64) |
| Positive predictive value | - | 0·57 (0·53-0·61) | 0·60 (0·56-0·63) | - | 0·61 (0·56-0·66) | 0·63 (0·59-0·66) | 0·64 (0·61-0·68) |
| Negative predictive value | - | 0·50 (0·45-0·55) | 0·53 (0·48-0·57) | - | 0·53 (0·48-0·58) | 0·56 (0·52-0·60) | 0·58 (0·54-0·62) |
| Likelihood ratio test p-value |  |  | <0·001 |  | <0·001 |  | 0·007 |

**Supplementary Table 11. Best-case sensitivity analysis for missing data age - model performance with and without GFAP**

| Metric | GFAP only | UPFRONT-ED | | HeadSMART | | CENTER-ED | |
| --- | --- | --- | --- | --- | --- | --- | --- |
|  |  | w/o GFAP | with GFAP | w/o GFAP | with GFAP | w/o GFAP | with GFAP |
| Area under the curve | 0·57 (0·53-0·61) | 0·56 (0·53-0·60) | 0·56 (0·53-0·60) | 0·57 (0·52-0·61) | 0·57 (0·53-0·62) | 0·67 (0·64-0·71) | 0·67 (0·64-0·71) |
| Variation explained (%) | 0 (-1-1) | 4 (1-7) | 4 (1-7) | 3 (0-6) | 3 (0-6) | 14 (9-19) | 14 (9-19) |
| Sensitivity | 0·00 (-0·02-0·02) | 0·12 (0·01-0·23) | 0·12 (0·01-0·23) | 0·10 (-0·05-0·25) | 0·10 (-0·04-0·25) | 0·32 (0·22-0·41) | 0·31 (0·22-0·40) |
| Specificity | 1·00 (0·98-1·01) | 0·93 (0·88-0·98) | 0·93 (0·87-0·98) | 0·94 (0·86-1·02) | 0·94 (0·86-1·01) | 0·87 (0·83-0·91) | 0·87 (0·83-0·91) |
| Positive predictive value | - | 0·51 (0·41-0·62) | 0·50 (0·40-0·60) | 0·53 (0·40-0·66) | 0·53 (0·41-0·65) | 0·59 (0·53-0·66) | 0·60 (0·53-0·66) |
| Negative predictive value | - | 0·63 (0·61-0·66) | 0·63 (0·60-0·66) | 0·63 (0·60-0·66) | 0·63 (0·60-0·66) | 0·68 (0·65-0·71) | 0·68 (0·65-0·71) |
| Likelihood ratio test p-value |  |  | 0·553 |  | 0·369 |  | 0·339 |

**Supplementary Table 12. Best-case sensitivity analysis for missing data age - model performance with and without NFL**

| Metric | NFL only | UPFRONT-ED | | HeadSMART | | CENTER-ED | |
| --- | --- | --- | --- | --- | --- | --- | --- |
|  |  | w/o NFL | with NFL | w/o NFL | with NFL | w/o NFL | with NFL |
| Area under the curve | 0·54 (0·50-0·58) | 0·56 (0·53-0·60) | 0·57 (0·53-0·60) | 0·57 (0·52-0·61) | 0·57 (0·53-0·62) | 0·67 (0·64-0·71) | 0·67 (0·64-0·71) |
| Variation explained (%) | 0 (-1-2) | 4 (1-7) | 4 (1-8) | 3 (0-6) | 4 (0-7) | 14 (9-19) | 14 (9-19) |
| Sensitivity | 0·01 (-0·02-0·03) | 0·12 (0·01-0·23) | 0·12 (0·01-0·23) | 0·10 (-0·05-0·25) | 0·10 (-0·04-0·24) | 0·32 (0·22-0·41) | 0·32 (0·22-0·41) |
| Specificity | 1·00 (0·99-1·00) | 0·93 (0·88-0·98) | 0·93 (0·88-0·98) | 0·94 (0·86-1·02) | 0·94 (0·86-1·01) | 0·87 (0·83-0·91) | 0·87 (0·83-0·90) |
| Positive predictive value | - | 0·51 (0·41-0·62) | 0·53 (0·43-0·63) | 0·53 (0·40-0·66) | 0·52 (0·39-0·65) | 0·59 (0·53-0·66) | 0·59 (0·53-0·66) |
| Negative predictive value | - | 0·63 (0·61-0·66) | 0·64 (0·61-0·67) | 0·63 (0·60-0·66) | 0·63 (0·60-0·66) | 0·68 (0·65-0·71) | 0·68 (0·65-0·71) |
| Likelihood ratio test p-value |  |  | 0·051 |  | 0·105 |  | 0·429 |

**Supplementary Table 13. Best-case sensitivity analysis for missing data age – model performance with and without S100B**

| Metric | S100B only | UPFRONT-ED | | HeadSMART | | CENTER-ED | |
| --- | --- | --- | --- | --- | --- | --- | --- |
|  |  | w/o S100B | with S100B | w/o S100B | with S100B | w/o S100B | with S100B |
| Area under the curve | 0·59 (0·55-0·62) | 0·56 (0·53-0·60) | 0·59 (0·56-0·63) | 0·57 (0·52-0·61) | 0·61 (0·57-0·65) | 0·67 (0·64-0·71) | 0·68 (0·64-0·71) |
| Variation explained (%) | 3 (1-5) | 4 (1-7) | 7 (3-10) | 3 (0-6) | 6 (2-10) | 14 (9-19) | 15 (10-20) |
| Sensitivity | 0·08 (0·03-0·12) | 0·12 (0·01-0·23) | 0·16 (0·06-0·27) | 0·10 (-0·05-0·25) | 0·15 (0·04-0·27) | 0·32 (0·22-0·41) | 0·31 (0·22-0·40) |
| Specificity | 0·96 (0·94-0·98) | 0·93 (0·88-0·98) | 0·92 (0·87-0·96) | 0·94 (0·86-1·02) | 0·92 (0·88-0·97) | 0·87 (0·83-0·91) | 0·86 (0·83-0·90) |
| Positive predictive value | 0·57 (0·44-0·69) | 0·51 (0·41-0·62) | 0·55 (0·47-0·63) | 0·53 (0·40-0·66) | 0·56 (0·46-0·65) | 0·59 (0·53-0·66) | 0·58 (0·51-0·64) |
| Negative predictive value | 0·63 (0·60-0·66) | 0·63 (0·61-0·66) | 0·64 (0·61-0·67) | 0·63 (0·60-0·66) | 0·64 (0·61-0·67) | 0·68 (0·65-0·71) | 0·67 (0·64-0·70) |
| Likelihood ratio test p-value |  |  | <0·001 |  | <0·001 |  | 0·006 |

**Supplementary Table 14. Best-case sensitivity analysis for missing data age - model performance with and without three serum biomarkers combined**

| Metric | GFAP, NFL & S100B only | UPFRONT-ED | | HeadSMART | | CENTER-ED | |
| --- | --- | --- | --- | --- | --- | --- | --- |
|  |  | w/o GFAP, NFL & S100B | with GFAP, NFL & S100B | w/o GFAP, NFL & S100B | with GFAP, NFL & S100B | w/o GFAP, NFL & S100B | with GFAP, NFL & S100B |
| Area under the curve | 0·58 (0·54-0·62) | 0·56 (0·53-0·60) | 0·59 (0·56-0·63) | 0·57 (0·52-0·61) | 0·61 (0·57-0·65) | 0·67 (0·64-0·71) | 0·68 (0·64-0·71) |
| Variation explained (%) | 3 (1-6) | 4 (1-7) | 7 (3-11) | 3 (0-6) | 6 (3-10) | 14 (9-19) | 15 (10-20) |
| Sensitivity | 0·09 (0·03-0·14) | 0·12 (0·01-0·23) | 0·17 (0·07-0·27) | 0·10 (-0·05-0·25) | 0·14 (0·03-0·26) | 0·32 (0·22-0·41) | 0·32 (0·23-0·40) |
| Specificity | 0·96 (0·94-0·98) | 0·93 (0·88-0·98) | 0·91 (0·87-0·96) | 0·94 (0·86-1·02) | 0·92 (0·87-0·97) | 0·87 (0·83-0·91) | 0·86 (0·83-0·90) |
| Positive predictive value | 0·58 (0·46-0·69) | 0·51 (0·41-0·62) | 0·55 (0·47-0·63) | 0·53 (0·40-0·66) | 0·54 (0·44-0·64) | 0·59 (0·53-0·66) | 0·59 (0·52-0·65) |
| Negative predictive value | 0·63 (0·61-0·66) | 0·63 (0·61-0·66) | 0·64 (0·62-0·67) | 0·63 (0·60-0·66) | 0·64 (0·61-0·67) | 0·68 (0·65-0·71) | 0·68 (0·65-0·71) |
| Likelihood ratio test p-value |  |  | <0·001 |  | <0·001 |  | 0·018 |

**Supplementary Table 15. Sensitivity analysis of NFL timing - model performance with and without subacute (~2-3 week) NFL**. In the CENTER-TBI study, the 2-3 week sampling time point was defined as 10-27 days. The table shows results for adults who sustained a mild TBI and had a normal CT as well as NFL sampled at 2-3 weeks, n = 529, median sample time 18 days (range 10-27 days) post-injury.

| Metric | NFL only | UPFRONT-ED | | HeadSMART | | CENTER-ED | |
| --- | --- | --- | --- | --- | --- | --- | --- |
|  |  | w/o NFL  (2-3 wks) | with NFL  (2-3 wks) | w/o NFL  (2-3 wks) | with NFL  (2-3 wks) | w/o NFL  (2-3 wks) | with NFL  (2-3 wks) |
| Area under the curve | 0.57 (0.52-0.63) | 0.58 (0.53-0.64) | 0.59 (0.54-0.65) | 0.59 (0.53-0.65) | 0.60 (0.54-0.66) | 0.70 (0.65-0.75) | 0.70 (0.66-0.75) |
| Variation explained (%) | 1 (-3-5) | 7 (1-13) | 8 (2-15) | 5 (0-10) | 6 (0-12) | 21 (12-29) | 21 (13-29) |
| Sensitivity | 0.94 (0.41-1.48) | 0.63 (0.45-0.81) | 0.62 (0.45-0.79) | 0.60 (0.28-0.93) | 0.61 (0.33-0.88) | 0.67 (0.58-0.76) | 0.67 (0.58-0.75) |
| Specificity | 0.07 (-0.55-0.68) | 0.49 (0.26-0.71) | 0.51 (0.29-0.72) | 0.51 (0.10-0.92) | 0.54 (0.18-0.89) | 0.63 (0.51-0.75) | 0.64 (0.51-0.76) |
| Positive predictive value | - | 0.59 (0.53-0.64) | 0.59 (0.53-0.65) | 0.60 (0.52-0.68) | 0.61 (0.54-0.68) | 0.68 (0.63-0.72) | 0.68 (0.63-0.73) |
| Negative predictive value | - | 0.53 (0.47-0.59) | 0.53 (0.46-0.60) | 0.53 (0.47-0.60) | 0.55 (0.49-0.61) | 0.62 (0.56-0.68) | 0.62 (0.57-0.68) |
| Likelihood ratio test p-value |  |  | 0.108 |  | 0.162 |  | 0.303 |

Supplementary Table 16. Characteristics of controls in the DTI cohort

|  | Overall (N=157) |
| --- | --- |
| **Age (years)** |  |
| median (min-max) | 39 (21 - 68) |
| **Sex** |  |
| female | 67 (43 %) |
| male | 89 (57 %) |
| missing | 1 (0·6%) |

Supplementary Table 17. Association of traumatic MRI abnormalities with outcome in univariable logistic regression analyses. Recovery refers to an extended Glasgow Outcome Scale = 8 at 3 months. OR = Odds ratio, 95% CI = 95% confidence interval. Note only patients with available outcome data were used here (n = 149).

|  | Recovery | |  |
| --- | --- | --- | --- |
| MRI abnormality | complete n=79 | incomplete n=70 | OR (95% CI) |
| any traumatic abnormality | 16 (20%) | 20 (29%) | 1·57 (0·69-3·61) |
| petechial haemorrhage | 10 (13%) | 16 (23%) | 2·03 (0·79-5·45) |
| subdural haemorrhage | 1 (1%) | 4 (6%) | 4·68 (0·45-235·44) |
| subarachnoid haemorrhage | 3 (4%) | 1 (1%) | 0·37 (0·01-4·72) |
| contusion | 2 (3%) | 3 (4%) | 1·72 (0·19-21·13) |
| intraventricular hemorrhage | 0 (0%) | 2 (3%) | Inf (0·21-Inf) |
| cisternal compression | 0 (0%) | 1 (1%) | Inf (0·03-Inf) |
| midline shift | 0 (0%) | 0 (0%) |  |
| extradural hemorrhage | 0 (0%) | 0 (0%) |  |

Supplementary Table 18. Coefficients for models with and without DTI. Model coefficients were pooled across the 10 imputed datasets. LL and UL = lower level and upper level of the 95% confidence interval. Sig = statistical significance recorded as either significant (signif) or not significant (ns).

| **Model** | **Variable** | **Pooled beta** | **LL** | **UL** | **Sig** |
| --- | --- | --- | --- | --- | --- |
| DTI only | (Intercept) | -8·029 | -10·798 | -5·259 | signif |
|  | DTI score | 16·252 | 10·713 | 21·791 | signif |
| UPFRONT-ED | (Intercept) | 0·648 | -1·248 | 2·544 | ns |
|  | Sex - male | -0·460 | -1·214 | 0·295 | ns |
|  | Education - diploma | -1·108 | -4·054 | 1·838 | ns |
|  | Education - university degree | -1·326 | -3·869 | 1·218 | ns |
|  | Age | -0·010 | -0·045 | 0·026 | ns |
|  | Mental health problems - present | 0·389 | -0·577 | 1·355 | ns |
|  | Alcohol intoxication - present | 0·308 | -0·724 | 1·341 | ns |
|  | GCS - 15 | -0·263 | -1·230 | 0·705 | ns |
|  | PTA over 1h - present | -0·668 | -1·673 | 0·338 | ns |
|  | Education - diploma:Age | 0·041 | -0·025 | 0·107 | ns |
|  | Education - university degree:Age | 0·036 | -0·020 | 0·092 | ns |
| UPFRONT-PLUS | (Intercept) | -0·910 | -2·257 | 0·437 | ns |
|  | Education - diploma | 0·673 | -0·407 | 1·752 | ns |
|  | Education - university degree | 0·193 | -0·673 | 1·060 | ns |
|  | Mental health problems - present | 0·065 | -1·045 | 1·174 | ns |
|  | Alcohol intoxication - present | 0·195 | -0·888 | 1·279 | ns |
|  | GCS - 15 | -0·051 | -1·086 | 0·984 | ns |
|  | PTA over 1h - present | -0·574 | -1·610 | 0·462 | ns |
|  | two week GAD7 score (anxiety) | 0·035 | -0·109 | 0·179 | ns |
|  | two week PHQ9 score (depression) | 0·054 | -0·041 | 0·149 | ns |
|  | two week RPQ score (concussion) | 0·020 | -0·042 | 0·083 | ns |
| HeadSMART | (Intercept) | -0·542 | -1·674 | 0·590 | ns |
|  | Age | 0·011 | -0·013 | 0·034 | ns |
|  | Mental health problems - present | 0·436 | -0·485 | 1·356 | ns |
|  | moderate-severe headache | -0·374 | -1·206 | 0·458 | ns |
|  | poor concentration | -0·241 | -1·012 | 0·529 | ns |
|  | light sensitivity | 0·453 | -0·366 | 1·272 | ns |
| CENTER-ED | (Intercept) | -0·870 | -2·598 | 0·859 | ns |
|  | Age | -0·002 | -0·030 | 0·025 | ns |
|  | ISS | 0·103 | 0·034 | 0·172 | signif |
|  | GCS - 15 | 0·151 | -0·788 | 1·091 | ns |
|  | Sex - male | -0·933 | -1·769 | -0·098 | signif |
|  | Mental health problems - present | 0·489 | -0·607 | 1·585 | ns |
|  | ASA - mild disease | 0·130 | -0·744 | 1·003 | ns |
|  | ASA - severe or life-threatening disease | 3·037 | 0·788 | 5·285 | signif |
|  | Cause of injury - traffic | 0·515 | -0·261 | 1·290 | ns |
|  | Cause of injury - violence | 0·424 | -1·079 | 1·928 | ns |
|  | acute RPQ score (concussion) | 0·003 | -0·034 | 0·039 | ns |
| CENTER-PLUS | (Intercept) | -2·245 | -4·388 | -0·101 | signif |
|  | Age | 0·012 | -0·013 | 0·038 | ns |
|  | ISS | 0·086 | 0·020 | 0·152 | signif |
|  | GCS - 15 | 0·167 | -0·817 | 1·152 | ns |
|  | Mental health problems - present | 0·353 | -0·693 | 1·399 | ns |
|  | Cause of injury - traffic | 0·216 | -0·534 | 0·965 | ns |
|  | Cause of injury - violence | 0·373 | -1·125 | 1·871 | ns |
|  | two week RPQ score (concussion) | 0·025 | -0·032 | 0·082 | ns |
|  | two week PCL5 score (PTSD) | 0·030 | -0·038 | 0·098 | ns |
| UPFRONT-ED with DTI | (Intercept) | -11·666 | -17·050 | -6·282 | signif |
|  | Sex - male | -0·011 | -1·301 | 1·279 | ns |
|  | Education - diploma | 3·940 | -1·387 | 9·267 | ns |
|  | Education - university degree | -0·299 | -4·882 | 4·283 | ns |
|  | Age | 0·058 | -0·005 | 0·121 | ns |
|  | Mental health problems - present | 0·630 | -1·154 | 2·413 | ns |
|  | Alcohol intoxication - present | -0·831 | -2·479 | 0·816 | ns |
|  | GCS - 15 | -0·181 | -1·884 | 1·521 | ns |
|  | PTA over 1h - present | -1·039 | -2·862 | 0·784 | ns |
|  | DTI score | 19·789 | 12·713 | 26·864 | signif |
|  | Education - diploma:Age | -0·073 | -0·186 | 0·039 | ns |
|  | Education - university degree:Age | -0·014 | -0·112 | 0·084 | ns |
| UPFRONT-PLUS with DTI | (Intercept) | -8·965 | -12·907 | -5·024 | signif |
|  | Education - diploma | 0·716 | -0·913 | 2·344 | ns |
|  | Education - university degree | -0·933 | -2·323 | 0·458 | ns |
|  | Mental health problems - present | -0·038 | -1·952 | 1·875 | ns |
|  | Alcohol intoxication - present | -0·912 | -2·494 | 0·670 | ns |
|  | GCS - 15 | 0·122 | -1·635 | 1·879 | ns |
|  | PTA over 1h - present | -0·960 | -2·785 | 0·865 | ns |
|  | two week GAD7 score (anxiety) | 0·087 | -0·110 | 0·284 | ns |
|  | two week PHQ9 score (depression) | 0·020 | -0·114 | 0·154 | ns |
|  | two week RPQ score (concussion) | 0·009 | -0·061 | 0·080 | ns |
|  | DTI score | 18·068 | 11·443 | 24·693 | signif |
| HeadSMART with DTI | (Intercept) | -9·872 | -13·610 | -6·135 | signif |
|  | Age | 0·030 | -0·012 | 0·072 | ns |
|  | Mental health problems - present | 0·531 | -1·061 | 2·124 | ns |
|  | moderate-severe headache | -1·084 | -2·483 | 0·314 | ns |
|  | poor concentration | -0·135 | -1·419 | 1·148 | ns |
|  | light sensitivity | -0·021 | -1·235 | 1·193 | ns |
|  | DTI score | 17·949 | 11·785 | 24·113 | signif |
| CENTER-ED with DTI | (Intercept) | -10·513 | -15·260 | -5·766 | signif |
|  | Age | 0·011 | -0·038 | 0·060 | ns |
|  | ISS | 0·100 | 0·001 | 0·200 | signif |
|  | GCS - 15 | 0·463 | -1·072 | 1·999 | ns |
|  | Sex - male | -0·079 | -1·350 | 1·193 | ns |
|  | Mental health problems - present | 0·212 | -1·600 | 2·024 | ns |
|  | ASA - mild disease | 1·076 | -0·518 | 2·670 | ns |
|  | ASA - severe or life-threatening disease | 2·402 | -0·521 | 5·325 | ns |
|  | Cause of injury - traffic | 0·138 | -1·153 | 1·429 | ns |
|  | Cause of injury - violence | -0·517 | -2·779 | 1·746 | ns |
|  | acute RPQ score (concussion) | -0·024 | -0·087 | 0·038 | ns |
|  | DTI score | 17·631 | 11·455 | 23·808 | signif |
| CENTER-PLUS with DTI | (Intercept) | -12·395 | -17·466 | -7·324 | signif |
|  | Age | 0·039 | -0·007 | 0·085 | ns |
|  | ISS | 0·071 | -0·025 | 0·167 | ns |
|  | GCS - 15 | 0·794 | -0·719 | 2·307 | ns |
|  | Mental health problems - present | 0·399 | -1·372 | 2·170 | ns |
|  | Cause of injury - traffic | -0·004 | -1·239 | 1·231 | ns |
|  | Cause of injury - violence | -0·771 | -3·080 | 1·538 | ns |
|  | two week RPQ score (concussion) | 0·009 | -0·066 | 0·084 | ns |
|  | two week PCL5 score (PTSD) | 0·047 | -0·041 | 0·134 | ns |
|  | DTI score | 17·927 | 11·490 | 24·363 | signif |

**Supplementary Table 19. Sensitivity analysis using DTI data not adjusted for age - model performance with and without DTI**

| Metric | DTI only | UPFRONT-ED | | UPFRONT-PLUS | | HeadSMART | | CENTER-ED | | CENTER-PLUS | |
| --- | --- | --- | --- | --- | --- | --- | --- | --- | --- | --- | --- |
|  |  | w/o DTI | with DTI | w/o DTI | with DTI | w/o DTI | with DTI | w/o DTI | with DTI | w/o DTI | with DTI |
| Area under the curve | 0·83 (0·80-0·86) | 0·57 (0·48-0·66) | 0·83 (0·81-0·86) | 0·64 (0·51-0·76) | 0·83 (0·81-0·86) | 0·53 (0·43-0·63) | 0·83 (0·80-0·86) | 0·67 (0·59-0·74) | 0·83 (0·81-0·86) | 0·68 (0·56-0·80) | 0·83 (0·81-0·86) |
| R^2^ (%) | 76 (62-90) | 10 (-4-23) | 81 (-9-100) | 17 (0-33) | 80 (16-100) | 4 (-5-13) | 78 (56-100) | 23 (9-37) | 80 (17-100) | 21 (4-39) | 79 (46-100) |
| Sensitivity | 0·74 (0·66-0·82) | 0·47 (0·25-0·68) | 0·77 (0·69-0·84) | 0·52 (0·28-0·77) | 0·76 (0·69-0·84) | 0·36 (0·04-0·68) | 0·74 (0·66-0·82) | 0·49 (0·30-0·67) | 0·75 (0·69-0·81) | 0·56 (0·37-0·74) | 0·76 (0·69-0·83) |
| Specificity | 0·75 (0·68-0·83) | 0·67 (0·49-0·84) | 0·78 (0·72-0·84) | 0·68 (0·50-0·85) | 0·78 (0·73-0·84) | 0·69 (0·38-1·01) | 0·76 (0·69-0·84) | 0·73 (0·59-0·87) | 0·76 (0·70-0·82) | 0·72 (0·57-0·88) | 0·77 (0·70-0·84) |
| Positive predictive value | 0·73 (0·64-0·81) | 0·56 (0·45-0·67) | 0·76 (0·69-0·82) | 0·59 (0·42-0·76) | 0·76 (0·70-0·82) | 0·51 (0·35-0·68) | 0·74 (0·66-0·82) | 0·62 (0·52-0·72) | 0·73 (0·67-0·80) | 0·64 (0·51-0·78) | 0·74 (0·67-0·82) |
| Negative predictive value | 0·76 (0·69-0·84) | 0·58 (0·49-0·67) | 0·79 (0·72-0·86) | 0·61 (0·48-0·75) | 0·79 (0·72-0·86) | 0·54 (0·44-0·64) | 0·77 (0·70-0·83) | 0·61 (0·52-0·70) | 0·77 (0·72-0·83) | 0·64 (0·54-0·75) | 0·78 (0·72-0·84) |
| Likelihood ratio test p-value |  |  | <0·001 |  | <0·001 |  | <0·001 |  | <0·001 |  | <0·001 |

**Supplementary Table 20. Worst-case sensitivity analysis for missing data age - model performance with and without DTI**

| Metric | DTI only | UPFRONT-ED | | UPFRONT-PLUS | | HeadSMART | | CENTER-ED | | CENTER-PLUS | |
| --- | --- | --- | --- | --- | --- | --- | --- | --- | --- | --- | --- |
|  |  | w/o DTI | with DTI | w/o DTI | with DTI | w/o DTI | with DTI | w/o DTI | with DTI | w/o DTI | with DTI |
| Area under the curve | 0·80 (0·76-0·83) | 0·57 (0·48-0·66) | 0·80 (0·78-0·83) | 0·62 (0·52-0·73) | 0·81 (0·78-0·84) | 0·52 (0·43-0·61) | 0·81 (0·78-0·83) | 0·66 (0·59-0·74) | 0·81 (0·79-0·83) | 0·64 (0·50-0·78) | 0·81 (0·78-0·84) |
| Variation explained (%) | 68 (52-83) | 9 (-4-22) | 73 (-43-100) | 15 (1-30) | 73 (-100-100) | 4 (-5-12) | 71 (52-90) | 22 (9-36) | 73 (33-100) | 17 (-2-35) | 73 (25-100) |
| Sensitivity | 0·68 (0·60-0·76) | 0·52 (0·31-0·73) | 0·71 (0·65-0·78) | 0·51 (0·30-0·73) | 0·72 (0·64-0·79) | 0·38 (0·06-0·70) | 0·70 (0·62-0·78) | 0·52 (0·34-0·69) | 0·68 (0·62-0·75) | 0·52 (0·30-0·74) | 0·71 (0·63-0·79) |
| Specificity | 0·67 (0·60-0·74) | 0·61 (0·42-0·80) | 0·70 (0·63-0·76) | 0·66 (0·46-0·85) | 0·72 (0·64-0·80) | 0·65 (0·32-0·98) | 0·70 (0·63-0·77) | 0·69 (0·54-0·84) | 0·73 (0·67-0·79) | 0·68 (0·51-0·84) | 0·71 (0·64-0·78) |
| Positive predictive value | 0·65 (0·58-0·73) | 0·55 (0·45-0·66) | 0·68 (0·61-0·75) | 0·59 (0·45-0·72) | 0·71 (0·63-0·79) | 0·52 (0·40-0·64) | 0·68 (0·61-0·76) | 0·61 (0·50-0·72) | 0·71 (0·65-0·78) | 0·60 (0·47-0·74) | 0·69 (0·62-0·77) |
| Negative predictive value | 0·69 (0·62-0·76) | 0·57 (0·48-0·67) | 0·73 (0·67-0·79) | 0·59 (0·49-0·69) | 0·74 (0·67-0·80) | 0·53 (0·44-0·62) | 0·72 (0·65-0·79) | 0·60 (0·51-0·70) | 0·71 (0·65-0·77) | 0·60 (0·48-0·72) | 0·73 (0·65-0·80) |
| Likelihood ratio test p-value |  |  | <0·001 |  | <0·001 |  | <0·001 |  | <0·001 |  | <0·001 |

**Supplementary 21. Best-case sensitivity analysis for missing data age - model performance with and without DTI**

| Metric | DTI only | UPFRONT-ED | | UPFRONT-PLUS | | HeadSMART | | CENTER-ED | | CENTER-PLUS | |
| --- | --- | --- | --- | --- | --- | --- | --- | --- | --- | --- | --- |
|  |  | w/o DTI | with DTI | w/o DTI | with DTI | w/o DTI | with DTI | w/o DTI | with DTI | w/o DTI | with DTI |
| Area under the curve | 0·80 (0·76-0·83) | 0·57 (0·49-0·65) | 0·80 (0·77-0·83) | 0·64 (0·52-0·76) | 0·81 (0·78-0·84) | 0·53 (0·44-0·62) | 0·80 (0·77-0·83) | 0·66 (0·58-0·73) | 0·80 (0·78-0·83) | 0·66 (0·52-0·79) | 0·81 (0·78-0·84) |
| Variation explained (%) | 70 (53-86) | 10 (-3-24) | 74 (30-100) | 17 (0-34) | 75 (49-100) | 4 (-5 -13) | 72 (50 -94) | 19 (5-34) | 73 (-100-100) | 18 (0-37) | 75 (-100-100) |
| Sensitivity | 0·69 (0·60-0·78) | 0·44 (0·22-0·65) | 0·70 (0·62-0·78) | 0·50 (0·24-0·75) | 0·69 (0·60-0·79) | 0·30 (-0·02-0·62) | 0·68 (0·59-0·77) | 0·44 (0·27-0·62) | 0·70 (0·62-0·78) | 0·49 (0·27-0·71) | 0·69 (0·60-0·77) |
| Specificity | 0·71 (0·64-0·78) | 0·70 (0·54-0·87) | 0·73 (0·66-0·79) | 0·71 (0·56-0·87) | 0·73 (0·67-0·79) | 0·76 (0·49-1·03) | 0·70 (0·64-0·77) | 0·76 (0·62-0·89) | 0·72 (0·66-0·78) | 0·72 (0·56-0·88) | 0·72 (0·65-0·79) |
| Positive predictive value | 0·66 (0·59-0·74) | 0·56 (0·45-0·67) | 0·68 (0·61-0·76) | 0·59 (0·44-0·75) | 0·68 (0·61-0·76) | 0·53 (0·39-0·67) | 0·66 (0·58-0·73) | 0·61 (0·50-0·73) | 0·68 (0·61-0·75) | 0·60 (0·43-0·77) | 0·67 (0·59-0·75) |
| Negative predictive value | 0·74 (0·67-0·81) | 0·59 (0·50-0·68) | 0·74 (0·68-0·81) | 0·62 (0·51-0·74) | 0·74 (0·67-0·82) | 0·56 (0·47-0·64) | 0·73 (0·66-0·80) | 0·61 (0·53-0·70) | 0·75 (0·68-0·81) | 0·62 (0·51-0·74) | 0·73 (0·66-0·81) |
| Likelihood ratio test p-value |  |  | <0·001 |  | <0·001 |  | <0·001 |  | <0·001 |  | <0·001 |

Supplementary Table 22. Sample timing and concentration of serum biomarkers. The two columns refer to patients with complete versus incomplete recovery three months post-injury.

|  | complete (N=60) | incomplete (N=47) |
| --- | --- | --- |
| **Time to sample taken in ED (hours)** |  |  |
| Median (Min-Max) | 8·4 (2 - 24) | 12 (2·4 - 24) |
| **GFAP (ng/ml)** |  |  |
| Median (Min-Max) | 0·59 (0 - 15·9) | 1·0 (0·1 - 8·7) |
| **NFL (pg/ml)** |  |  |
| Median (Min-Max) | 5·9 (1·4 - 170·9) | 6·9 (1·3 - 18·7) |
| **S100B (ng/ml)** |  |  |
| Median (Min-Max) | 0·06 (0 - 0·3) | 0·08 (0 - 1·4) |

Supplementary Table 23. Using biomarkers to identify patients for DTI, using a range of minimum sensitivities. Biomarker cutoff levels were chosen assuming a range of minimum sensitivities (T). Avoided MRIs refers to the number of patients which would not be scanned due to their biomarker level falling below the cutoff threshold and who did go on to make a complete recovery (true negatives). Unnecessary MRIs refer to the number of patients who would have been scanned as their biomarker value exceeds the cutoff threshold but who would go on to make a complete recovery (false positives, FP). Missed incomplete recoveries refers to the number of patients who would not be scanned as their biomarker value falls below the cutoff threshold but who nonetheless would not recover completely (false negatives, FN).

| Sample | Time | T | N | Cutoff | Sens | Spec | Avoided MRIs | FP | FN | Number needed to scan |
| --- | --- | --- | --- | --- | --- | --- | --- | --- | --- | --- |
| GFAP | <12h | 1 | 63 | 0·06 | 1·00 (0·76-1·00) | 0·19 (0·10-0·35) | 7 (11%) | 30 (48%) | 0 (0%) | 2.2 |
| GFAP | 12-24h | 1 | 44 | 0·07 | 1·00 (0·72-1·00) | 0·13 (0·05-0·35) | 3 (7%) | 20 (45%) | 0 (0%) | 2.0 |
| GFAP | <12h | 0·95 | 63 | 0·12 | 0·96 (0·77-0·99) | 0·24 (0·14-0·41) | 10 (16%) | 28 (44%) | 1 (2%) | 2.1 |
| GFAP | 12-24h | 0·95 | 44 | 0·09 | 0·95 (0·72-0·99) | 0·22 (0·10-0·44) | 6 (14%) | 18 (41%) | 1 (2%) | 1.9 |
| GFAP | <12h | 0·9 | 63 | 0·13 | 0·92 (0·73-0·97) | 0·30 (0·18-0·46) | 13 (21%) | 26 (41%) | 2 (3%) | 2.1 |
| GFAP | 12-24h | 0·9 | 44 | 0·09 | 0·95 (0·72-0·99) | 0·22 (0·10-0·44) | 6 (14%) | 18 (41%) | 1 (2%) | 1.9 |
| GFAP | <12h | 0·8 | 63 | 0·34 | 0·88 (0·69-0·95) | 0·49 (0·33-0·64) | 21 (33%) | 19 (30%) | 3 (5%) | 1.9 |
| GFAP | 12-24h | 0·8 | 44 | 0·40 | 0·81 (0·58-0·92) | 0·35 (0·19-0·56) | 12 (27%) | 15 (34%) | 4 (9%) | 1.9 |
| S100B | <12h | 1 | 63 | 0·04 | 1·00 (0·76-1·00) | 0·11 (0·05-0·26) | 4 (6%) | 33 (52%) | 0 (0%) | 2.3 |
| S100B | 12-24h | 1 | 44 | 0·03 | 1·00 (0·72-1·00) | 0·04 (0·01-0·26) | 1 (2%) | 22 (50%) | 0 (0%) | 2.1 |
| S100B | <12h | 0·95 | 63 | 0·04 | 1·00 (0·76-1·00) | 0·11 (0·05-0·26) | 4 (6%) | 33 (52%) | 0 (0%) | 2.3 |
| S100B | 12-24h | 0·95 | 44 | 0·03 | 1·00 (0·72-1·00) | 0·04 (0·01-0·26) | 1 (2%) | 22 (50%) | 0 (0%) | 2.1 |
| S100B | <12h | 0·9 | 63 | 0·04 | 1·00 (0·76-1·00) | 0·11 (0·05-0·26) | 4 (6%) | 33 (52%) | 0 (0%) | 2.3 |
| S100B | 12-24h | 0·9 | 44 | 0·03 | 1·00 (0·72-1·00) | 0·04 (0·01-0·26) | 1 (2%) | 22 (50%) | 0 (0%) | 2.1 |
| S100B | <12h | 0·8 | 63 | 0·06 | 0·81 (0·61-0·91) | 0·32 (0·20-0·49) | 17 (27%) | 25 (40%) | 5 (8%) | 2.0 |
| S100B | 12-24h | 0·8 | 44 | 0·03 | 1·00 (0·72-1·00) | 0·04 (0·01-0·26) | 1 (2%) | 22 (50%) | 0 (0%) | 2.1 |
| NFL | <12h | 1 | 63 | 1·34 | 1·00 (0·76-1·00) | 0·00 (0·00-0·18) | 0 (0%) | 37 (59%) | 0 (0%) | 2.5 |
| NFL | 12-24h | 1 | 44 | 3·71 | 1·00 (0·72-1·00) | 0·13 (0·05-0·35) | 3 (7%) | 20 (45%) | 0 (0%) | 2.0 |
| NFL | <12h | 0·95 | 63 | 1·88 | 0·96 (0·77- 0·99) | 0·03 (0·01- 0·17) | 2 (3%) | 36 (57%) | 1 (2%) | 2.5 |
| NFL | 12-24h | 0·95 | 44 | 3·71 | 1·00 (0·72-1·00) | 0·13 (0·05-0·35) | 3 (7%) | 20 (45%) | 0 (0%) | 2.0 |
| NFL | <12h | 0·9 | 63 | 2·56 | 0·92 (0·73-0·97) | 0·08 (0·03-0·23) | 5 (8%) | 34 (54%) | 2 (3%) | 2.5 |
| NFL | 12-24h | 0·9 | 44 | 5·41 | 0·90 (0·68-0·97) | 0·35 (0·19-0·56) | 10 (23%) | 15 (34%) | 2 (5%) | 1.8 |
| NFL | <12h | 0·8 | 63 | 3·92 | 0·85 (0·65-0·93) | 0·38 (0·24-0·54) | 18 (29%) | 23 (37%) | 4 (6%) | 2.1 |
| NFL | 12-24h | 0·8 | 44 | 5·41 | 0·90 (0·68-0·97) | 0·35 (0·19-0·56) | 10 (23%) | 15 (34%) | 2 (5%) | 1.8 |

**Supplementary Table 24. Sensitivity analysis using only patients in the overlap cohort (n = 108) – model performance with and without GFAP**

| Metric | GFAP only | UPFRONT-ED | | HeadSMART | | CENTER-ED | |
| --- | --- | --- | --- | --- | --- | --- | --- |
|  |  | w/o GFAP | with GFAP | w/o GFAP | with GFAP | w/o GFAP | with GFAP |
| Area under the curve | 0·64 (0·51-0·76) | 0·62 (0·53-0·71) | 0·62 (0·52-0·71) | 0·61 (0·50-0·73) | 0·64 (0·53-0·75) | 0·68 (0·59-0·76) | 0·67 (0·58-0·75) |
| Variation explained (%) | 1 (-7-8) | 20 (3-37) | 20 (2-39) | 13 (-3-29) | 14 (-4-33) | 26 (8-43) | 26 (8-44) |
| Sensitivity | 0·04 (-0·43-0·50) | 0·42 (0·19-0·64) | 0·44 (0·21-0·67) | 0·35 (0·08-0·63) | 0·40 (0·13-0·67) | 0·49 (0·28-0·71) | 0·50 (0·29-0·70) |
| Specificity | 0·93 (0·51-1·35) | 0·73 (0·56-0·90) | 0·69 (0·54-0·85) | 0·86 (0·65-1·07) | 0·81 (0·65-0·98) | 0·72 (0·59-0·86) | 0·72 (0·59-0·85) |
| Positive predictive value | - | 0·56 (0·42-0·69) | 0·54 (0·40-0·68) | 0·68 (0·49-0·87) | 0·64 (0·46-0·82) | 0·59 (0·44-0·73) | 0·58 (0·44-0·72) |
| Negative predictive value | - | 0·61 (0·51-0·71) | 0·61 (0·50-0·72) | 0·63 (0·52-0·73) | 0·63 (0·53-0·73) | 0·64 (0·55-0·74) | 0·64 (0·54-0·74) |
| Likelihood ratio test p-value |  |  | 0·404 |  | 0·220 |  | 0·566 |

**Supplementary Table 25. Sensitivity analysis using only patients in the overlap cohort (n = 108) – model performance with and without NFL**

| Metric | NFL only | UPFRONT-ED | | HeadSMART | | CENTER-ED | |
| --- | --- | --- | --- | --- | --- | --- | --- |
|  |  | w/o NFL | with NFL | w/o NFL | with NFL | w/o NFL | with NFL |
| Area under the curve | 0·61 (0·49-0·74) | 0·62 (0·53-0·71) | 0·62 (0·52-0·71) | 0·61 (0·50-0·73) | 0·60 (0·49-0·72) | 0·68 (0·59-0·76) | 0·67 (0·58-0·75) |
| Variation explained (%) | 0 (-6-6) | 20 (3-37) | 20 (2-38) | 13 (-3-29) | 13 (-3-29) | 26 (8-43) | 26 (8-44) |
| Sensitivity | 0·00 (-0·54-0·53) | 0·42 (0·19-0·64) | 0·42 (0·19-0·65) | 0·35 (0·08-0·63) | 0·36 (0·08-0·63) | 0·49 (0·28-0·71) | 0·49 (0·28-0·70) |
| Specificity | 0·99 (0·49-1·49) | 0·73 (0·56-0·90) | 0·72 (0·55-0·89) | 0·86 (0·65-1·07) | 0·85 (0·65-1·05) | 0·72 (0·59-0·86) | 0·73 (0·60-0·86) |
| Positive predictive value | - | 0·56 (0·42-0·69) | 0·55 (0·42-0·69) | 0·68 (0·49-0·87) | 0·67 (0·49-0·85) | 0·59 (0·44-0·73) | 0·59 (0·45-0·74) |
| Negative predictive value | - | 0·61 (0·51-0·71) | 0·61 (0·50-0·71) | 0·63 (0·52-0·73) | 0·62 (0·52-0·73) | 0·64 (0·55-0·74) | 0·64 (0·54-0·74) |
| Likelihood ratio test p-value |  |  | 0·456 |  | 0·758 |  | 0·520 |

**Supplementary Table 26. Sensitivity analysis using only patients in the overlap cohort (n = 108) – model performance with and without S100B**

| Metric | S100B only | UPFRONT-ED | | HeadSMART | | CENTER-ED | |
| --- | --- | --- | --- | --- | --- | --- | --- |
|  |  | w/o S100B | with S100B | w/o S100B | with S100B | w/o S100B | with S100B |
| Area under the curve | 0·60 (0·50-0·71) | 0·62 (0·53-0·71) | 0·67 (0·58-0·75) | 0·61 (0·50-0·73) | 0·67 (0·57-0·77) | 0·68 (0·59-0·76) | 0·68 (0·61-0·76) |
| Variation explained (%) | 7 (-2-17) | 20 (3-37) | 28 (10-46) | 13 (-3-29) | 20 (3-37) | 26 (8-43) | 30 (13-47) |
| Sensitivity | 0·25 (-0·03-0·53) | 0·42 (0·19-0·64) | 0·51 (0·31-0·71) | 0·35 (0·08-0·63) | 0·47 (0·26-0·69) | 0·49 (0·28-0·71) | 0·49 (0·28-0·69) |
| Specificity | 0·85 (0·61-1·10) | 0·73 (0·56-0·90) | 0·76 (0·60-0·91) | 0·86 (0·65-1·07) | 0·79 (0·64-0·94) | 0·72 (0·59-0·86) | 0·70 (0·57-0·84) |
| Positive predictive value | - | 0·56 (0·42-0·69) | 0·63 (0·49-0·76) | 0·68 (0·49-0·87) | 0·64 (0·48-0·80) | 0·59 (0·44-0·73) | 0·57 (0·43-0·71) |
| Negative predictive value | - | 0·61 (0·51-0·71) | 0·66 (0·55-0·77) | 0·63 (0·52-0·73) | 0·65 (0·55-0·76) | 0·64 (0·55-0·74) | 0·63 (0·53-0·73) |
| Likelihood ratio test p-value |  |  | 0·005 |  | 0·011 |  | 0·041 |

**Supplementary Table 27. Sensitivity analysis using only patients in the overlap cohort (n = 108) – model performance with and without all three biomarkers combined**

| Metric | GFAP, NFL & S100B only | UPFRONT-ED | | HeadSMART | | CENTER-ED | |
| --- | --- | --- | --- | --- | --- | --- | --- |
|  |  | w/o GFAP, NFL & S100B | with GFAP, NFL & S100B | w/o GFAP, NFL & S100B | with GFAP, NFL & S100B | w/o GFAP, NFL & S100B | with GFAP, NFL & S100B |
| Area under the curve | 0·57 (0·44-0·69) | 0·62 (0·53-0·71) | 0·67 (0·58-0·75) | 0·61 (0·50-0·73) | 0·68 (0·58-0·77) | 0·68 (0·59-0·76) | 0·68 (0·61-0·76) |
| Variation explained (%) | 8 (-3-19) | 20 (3-37) | 30 (11-49) | 13 (-3-29) | 22 (4-40) | 26 (8-43) | 31 (14-49) |
| Sensitivity | 0·28 (0·00-0·56) | 0·42 (0·19-0·64) | 0·50 (0·30-0·69) | 0·35 (0·08-0·63) | 0·47 (0·24-0·70) | 0·49 (0·28-0·71) | 0·54 (0·33-0·74) |
| Specificity | 0·81 (0·60-1·01) | 0·73 (0·56-0·90) | 0·69 (0·55-0·83) | 0·86 (0·65-1·07) | 0·77 (0·63-0·91) | 0·72 (0·59-0·86) | 0·69 (0·57-0·82) |
| Positive predictive value | 0·53 (0·35-0·71) | 0·56 (0·42-0·69) | 0·56 (0·43-0·70) | 0·68 (0·49-0·87) | 0·62 (0·47-0·77) | 0·59 (0·44-0·73) | 0·58 (0·45-0·71) |
| Negative predictive value | 0·59 (0·49-0·69) | 0·61 (0·51-0·71) | 0·64 (0·53-0·74) | 0·63 (0·52-0·73) | 0·65 (0·54-0·75) | 0·64 (0·55-0·74) | 0·66 (0·56-0·75) |
| Likelihood ratio test p-value |  |  | 0·017 |  | 0·033 |  | 0·122 |

**Supplementary Table 28. Sensitivity analysis using only patients in the overlap cohort (n = 108) – model performance with and without DTI**

| Metric | DTI only | UPFRONT-ED | | UPFRONT-PLUS | | HeadSMART | | CENTER-ED | | CENTER-PLUS | |
| --- | --- | --- | --- | --- | --- | --- | --- | --- | --- | --- | --- |
|  |  | w/o DTI | with DTI | w/o DTI | with DTI | w/o DTI | with DTI | w/o DTI | with DTI | w/o DTI | with DTI |
| Area under the curve | 0·80 (0·75-0·84) | 0·62 (0·52-0·73) | 0·81 (0·78-0·84) | 0·67 (0·56-0·78) | 0·82 (0·79-0·85) | 0·60 (0·48-0·72) | 0·81 (0·77-0·84) | 0·68 (0·59-0·78) | 0·83 (0·80-0·86) | 0·70 (0·61-0·80) | 0·82 (0·79-0·85) |
| Variation explained (%) | 72 (52-91) | 21 (2-40) | 79 (-100-100) | 27 (7-46) | 78 (-100-100) | 12 (-4-28) | 75 (58-92) | 27 (8-45) | 81 (-79-100) | 28 (9-47) | 78 (62-95) |
| Sensitivity | 0·63 (0·50-0·76) | 0·44 (0·20-0·67) | 0·70 (0·62-0·79) | 0·47 (0·22-0·72) | 0·70 (0·60-0·80) | 0·33 (0·05-0·61) | 0·65 (0·54-0·75) | 0·48 (0·28-0·69) | 0·70 (0·57-0·82) | 0·54 (0·33-0·76) | 0·67 (0·58-0·76) |
| Specificity | 0·75 (0·67-0·83) | 0·74 (0·60-0·88) | 0·75 (0·70-0·81) | 0·73 (0·58-0·87) | 0·77 (0·72-0·82) | 0·85 (0·66-1·04) | 0·74 (0·66-0·82) | 0·71 (0·59-0·83) | 0·79 (0·73-0·86) | 0·74 (0·61-0·87) | 0·76 (0·70-0·82) |
| Positive predictive value | 0·68 (0·57-0·78) | 0·59 (0·45-0·72) | 0·69 (0·62-0·76) | 0·58 (0·43-0·74) | 0·71 (0·64-0·77) | 0·66 (0·48-0·83) | 0·67 (0·57-0·76) | 0·57 (0·45-0·70) | 0·73 (0·65-0·82) | 0·63 (0·50-0·75) | 0·70 (0·62-0·77) |
| Negative predictive value | 0·72 (0·63-0·81) | 0·62 (0·52-0·72) | 0·77 (0·71-0·83) | 0·63 (0·50-0·76) | 0·77 (0·70-0·84) | 0·61 (0·50-0·72) | 0·73 (0·65-0·81) | 0·63 (0·54-0·73) | 0·77 (0·68-0·85) | 0·67 (0·57-0·77) | 0·75 (0·68-0·81) |
| Likelihood ratio test p-value |  |  | <0·001 |  | <0·001 |  | <0·001 |  | <0·001 |  | <0·001 |


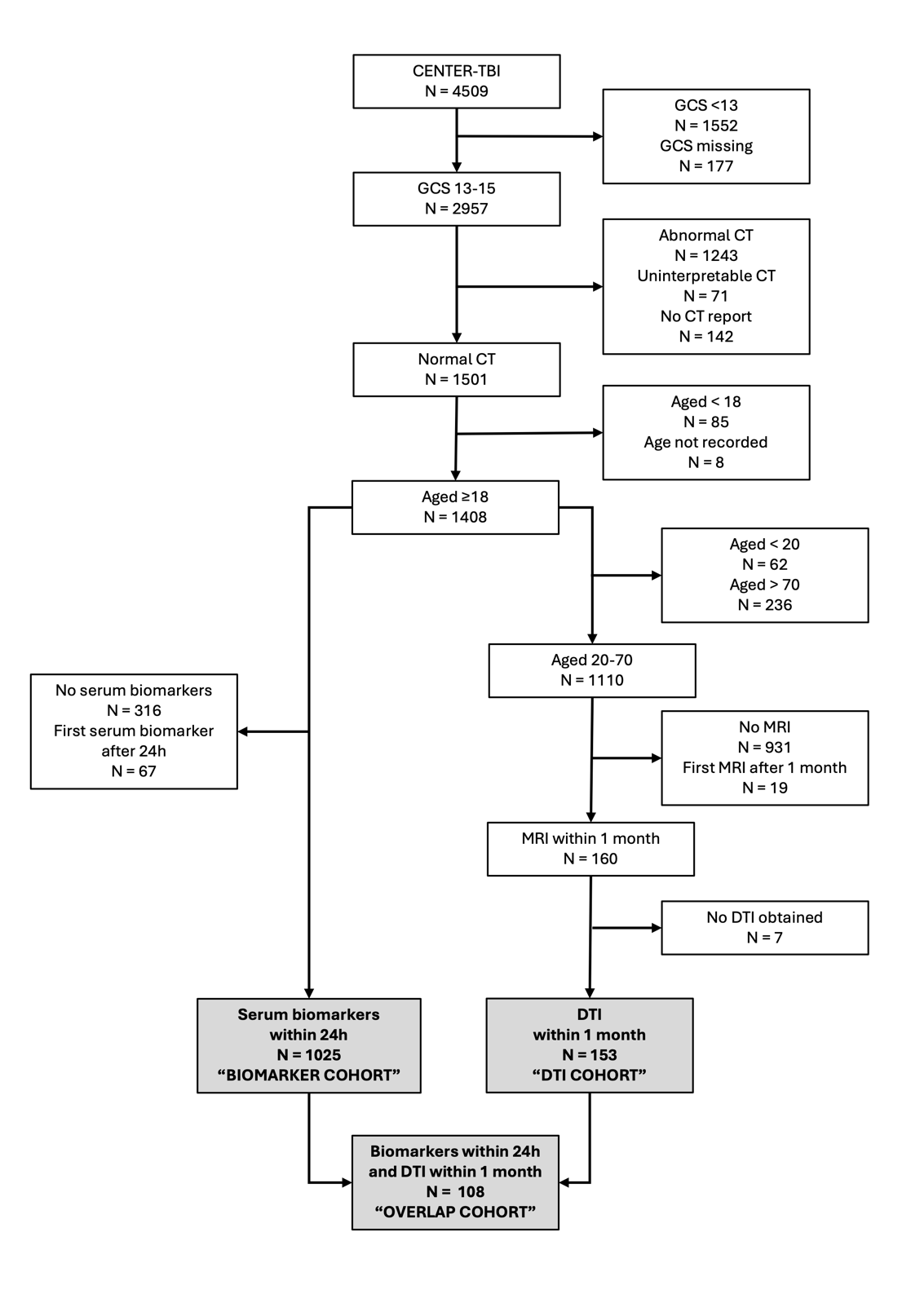


Supplementary Figure 1. Flowchart of patient inclusion. CT = computed tomography, MRI = magnetic resonance imaging, DTI = diffusion tensor imaging.


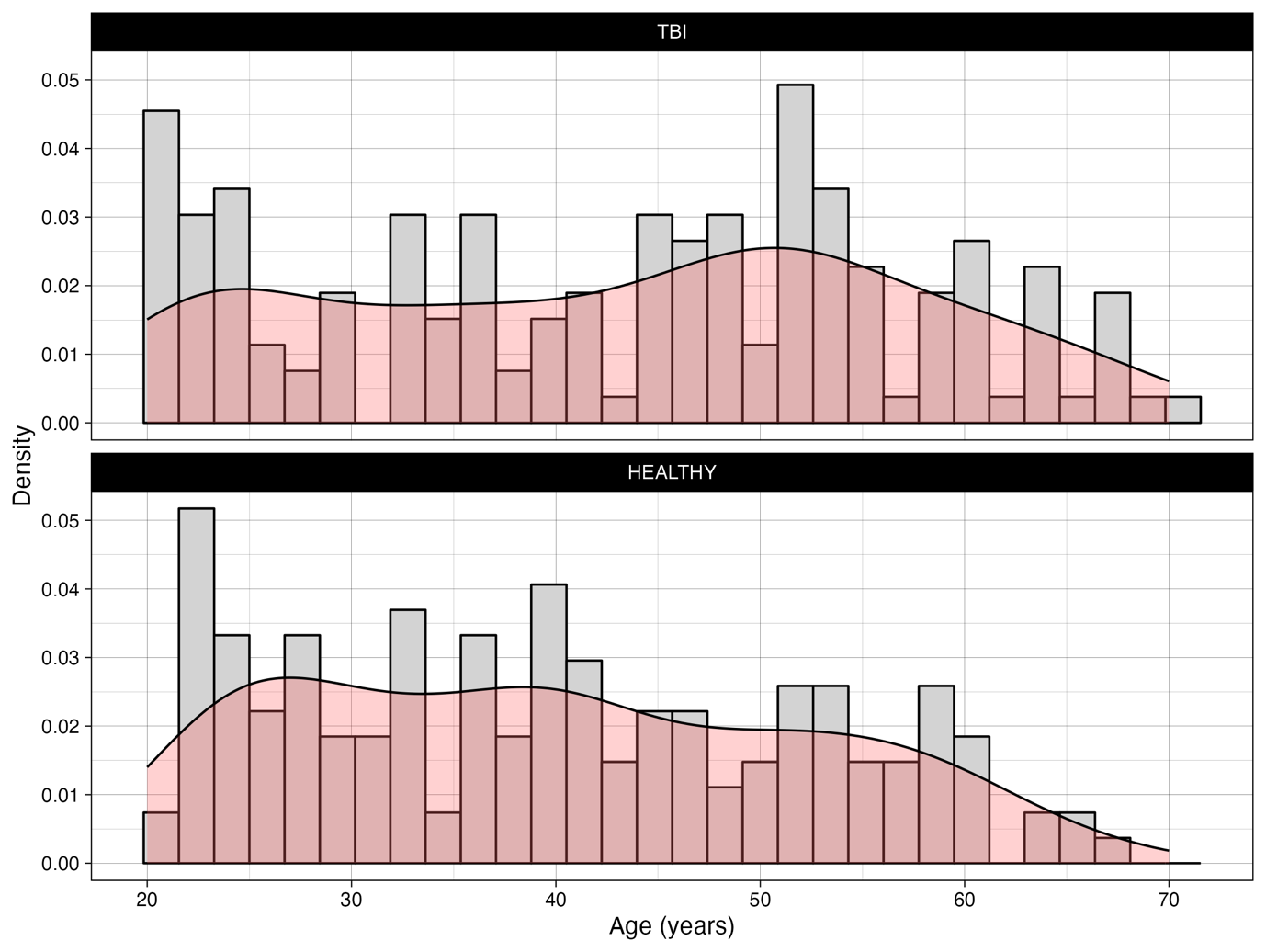


Supplementary Figure 2. Age distribution of the patients with mTBI and healthy volunteers for the participants who were included in the DTI analysis.


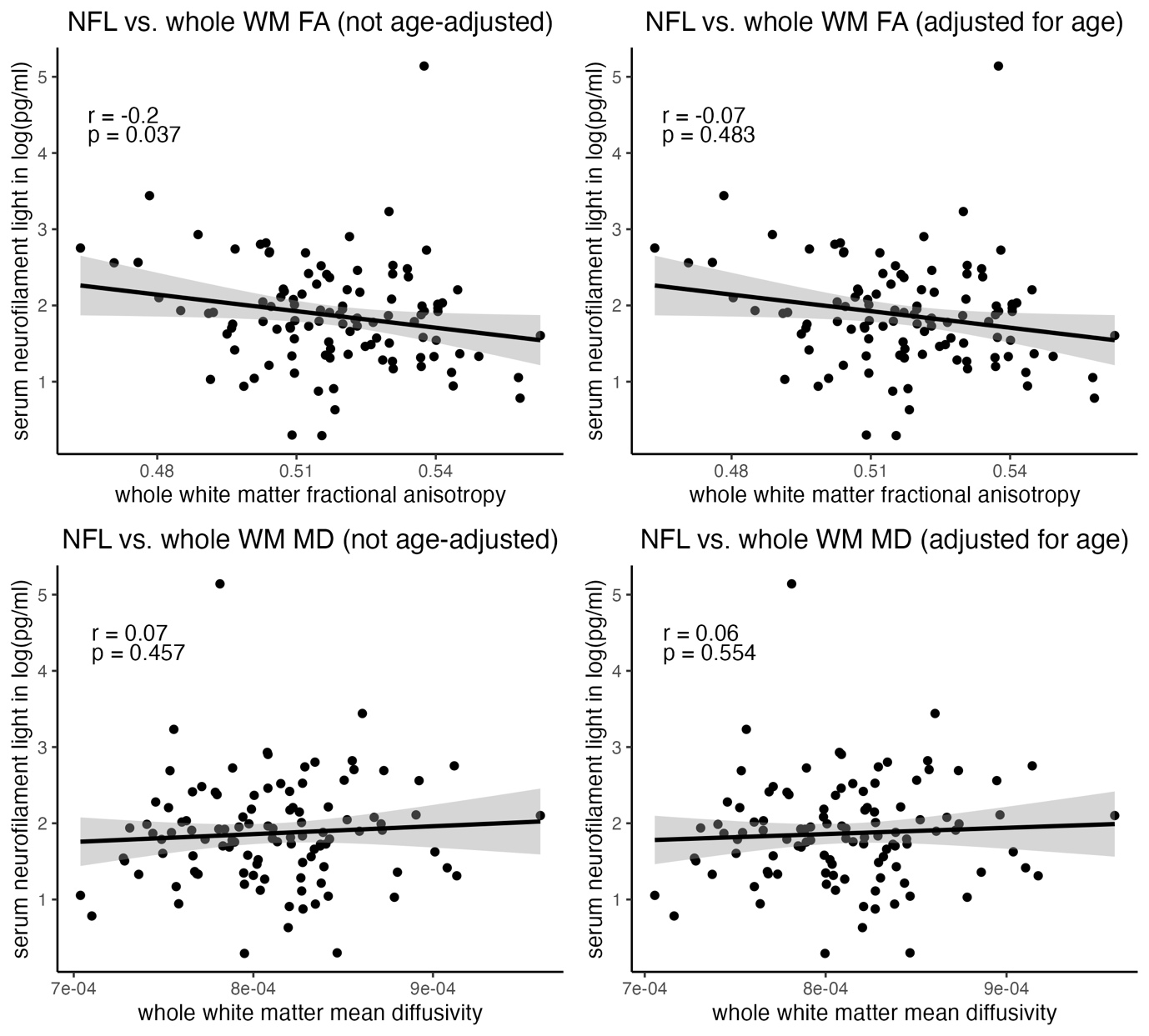


**Supplementary Figure 3. Association of serum neurofilament light (NFL) with diffusion metrics**


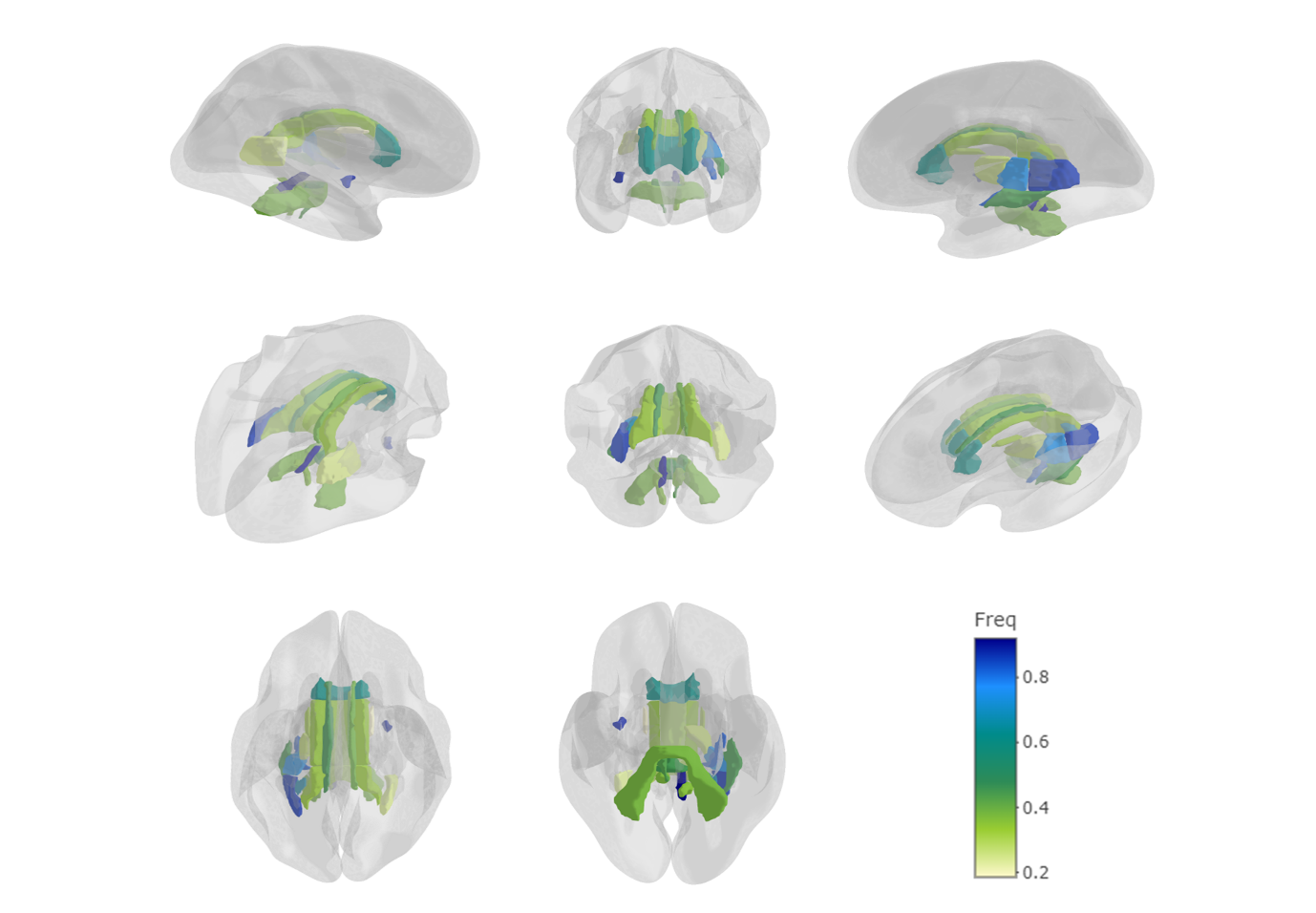


Supplementary Figure 4. Prognostic value of different white matter tracts. The figure shows those tracts from JHU ICBM-DTI-81 atlas which were identified as prognostic based on lasso regression. Tracts are colour-coded for their prognostic value determined by the frequency of selection, i.e., the proportion of times the tract was selected as prognostically relevant in the 2000 runs that comprised the analysis (200 bootstrap samples x 10 multiply imputed datasets). The views are from left to right by row: right, anterior, left, right-posterior, posterior, left-anterior, superior, inferior.


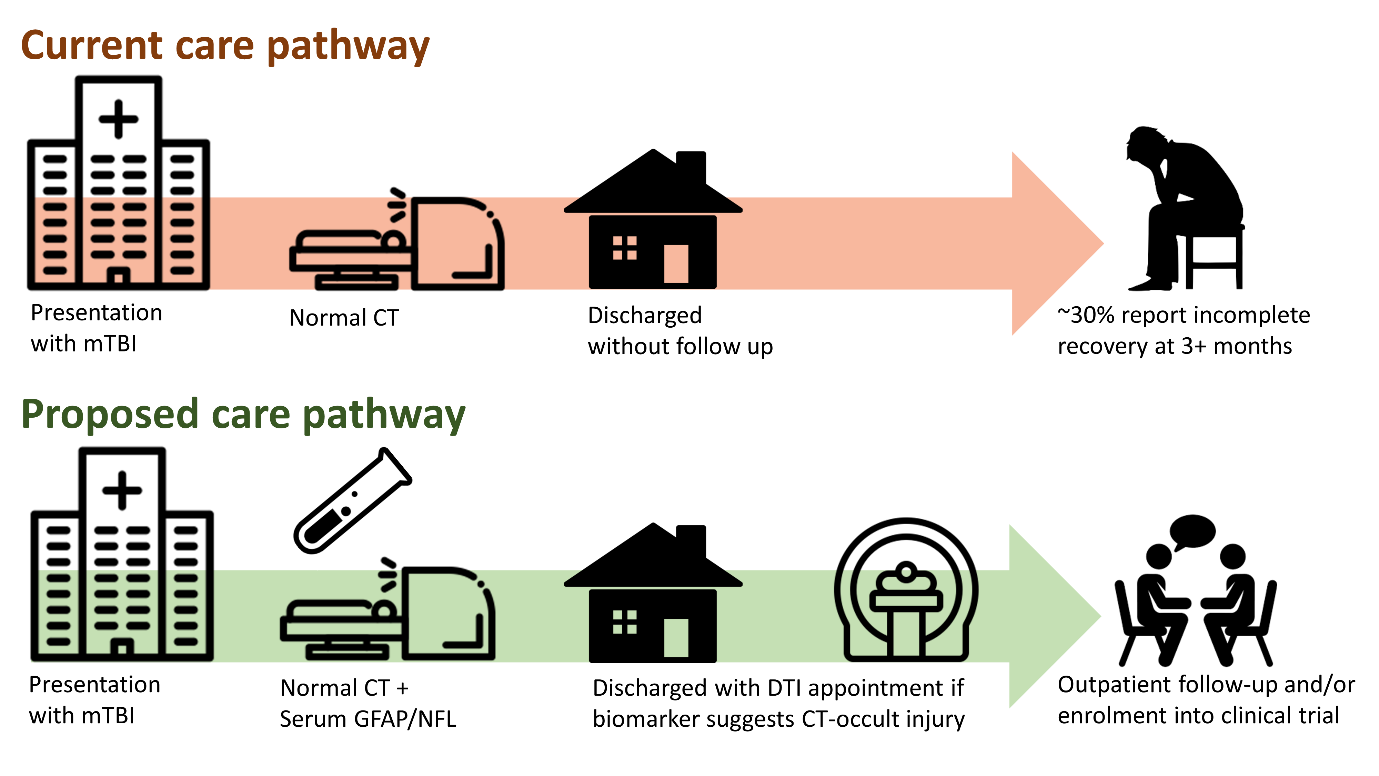


Supplementary Figure 5. Current and proposed care pathway based on findings of this study.

Supplementary Checklist – TRIPOD Checklist: Prediction Model Development and Validation

| **Section/Topic** | **Item** |  | **Checklist Item** | **Manuscript Page** |
| --- | --- | --- | --- | --- |
| **Title and abstract** | | | | |
| Title | 1 | D;V | Identify the study as developing and/or validating a multivariable prediction model, the target population, and the outcome to be predicted. | 1 |
| Abstract | 2 | D;V | Provide a summary of objectives, study design, setting, participants, sample size, predictors, outcome, statistical analysis, results, and conclusions. | 3 |
| **Introduction** | | | | |
| Background and objectives | 3a | D;V | Explain the medical context (including whether diagnostic or prognostic) and rationale for developing or validating the multivariable prediction model, including references to existing models. | 4-7 |
|  | 3b | D;V | Specify the objectives, including whether the study describes the development or validation of the model or both. | 7 |
| **Methods** | | | | |
| Source of data | 4a | D;V | Describe the study design or source of data (e.g., randomized trial, cohort, or registry data), separately for the development and validation data sets, if applicable. | 7 |
|  | 4b | D;V | Specify the key study dates, including start of accrual; end of accrual; and, if applicable, end of follow-up. | 7 |
| Participants | 5a | D;V | Specify key elements of the study setting (e.g., primary care, secondary care, general population) including number and location of centres. | 7 |
|  | 5b | D;V | Describe eligibility criteria for participants. | 7 |
|  | 5c | D;V | Give details of treatments received, if relevant. |  |
| Outcome | 6a | D;V | Clearly define the outcome that is predicted by the prediction model, including how and when assessed. | 7-10  Suppl. Methods 1 |
|  | 6b | D;V | Report any actions to blind assessment of the outcome to be predicted. | 7-10 |
| Predictors | 7a | D;V | Clearly define all predictors used in developing or validating the multivariable prediction model, including how and when they were measured. | 7-10 |
|  | 7b | D;V | Report any actions to blind assessment of predictors for the outcome and other predictors. | 7-10 |
| Sample size | 8 | D;V | Explain how the study size was arrived at. | 7 |
| Missing data | 9 | D;V | Describe how missing data were handled (e.g., complete-case analysis, single imputation, multiple imputation) with details of any imputation method. | 9 |
| Statistical analysis methods | 10a | D | Describe how predictors were handled in the analyses. | 8-10 |
|  | 10b | D | Specify type of model, all model-building procedures (including any predictor selection), and method for internal validation. | 9-10  Suppl. Methods 2 |
|  | 10c | V | For validation, describe how the predictions were calculated. | 9-10 |
|  | 10d | D;V | Specify all measures used to assess model performance and, if relevant, to compare multiple models. | 9-10 |
|  | 10e | V | Describe any model updating (e.g., recalibration) arising from the validation, if done. | 9,10 |
| Risk groups | 11 | D;V | Provide details on how risk groups were created, if done. | 9,10 |
| Development vs. validation | 12 | V | For validation, identify any differences from the development data in setting, eligibility criteria, outcome, and predictors. | 9,10 |
| **Results** | | | | |
| Participants | 13a | D;V | Describe the flow of participants through the study, including the number of participants with and without the outcome and, if applicable, a summary of the follow-up time. A diagram may be helpful. | 10  Supp. Figure 1 |
|  | 13b | D;V | Describe the characteristics of the participants (basic demographics, clinical features, available predictors), including the number of participants with missing data for predictors and outcome. | 10  Supp. Tables 1, 2 |
|  | 13c | V | For validation, show a comparison with the development data of the distribution of important variables (demographics, predictors and outcome). | 10  Suppl. Fig 2 |
| Model development | 14a | D | Specify the number of participants and outcome events in each analysis. | 10-11 |
|  | 14b | D | If done, report the unadjusted association between each candidate predictor and outcome. | 10  Table 3 |
| Model specification | 15a | D | Present the full prediction model to allow predictions for individuals (i.e., all regression coefficients, and model intercept or baseline survival at a given time point). | 10-11  Supp. Tables 3-15, 18-28 |
|  | 15b | D | Explain how to the use the prediction model. | 10-11  Table 5 |
| Model performance | 16 | D;V | Report performance measures (with CIs) for the prediction model. | 10-11  Table 2,  Figures 1 and 2, Supp. Tables 3-15, 18-28 |
| Model-updating | 17 | V | If done, report the results from any model updating (i.e., model specification, model performance). | 10-11 |
| **Discussion** | | | | |
| Limitations | 18 | D;V | Discuss any limitations of the study (such as nonrepresentative sample, few events per predictor, missing data). | 13-14 |
| Interpretation | 19a | V | For validation, discuss the results with reference to performance in the development data, and any other validation data. | 10-13 |
|  | 19b | D;V | Give an overall interpretation of the results, considering objectives, limitations, results from similar studies, and other relevant evidence. | 12-13 |
| Implications | 20 | D;V | Discuss the potential clinical use of the model and implications for future research. | 13-14 |
| **Other information** | | | | |
| Supplementary information | 21 | D;V | Provide information about the availability of supplementary resources, such as study protocol, Web calculator, and data sets. | 16 |
| Funding | 22 | D;V | Give the source of funding and the role of the funders for the present study. | 16 |
