# Supplementary material for: Predicting recovery in patients with mild traumatic brain injury and a normal CT using serum biomarkers and diffusion tensor imaging (CENTER-TBI): an observational cohort study
Source: eClinicalMedicine. 2024 Aug 8;75:102751. doi: 10.1016/j.eclinm.2024.102751 (PMC11667275; doi:10.1016/j.eclinm.2024.102751)
Supplement: Supplement Part 2 [file mmc2.docx]

**The Collaborative European NeuroTrauma Effectiveness Research in Traumatic Brain Injury Magnetic Resonance Imaging (CENTER-TBI MRI) Sub-study Participants and Investigators**

| **First Name** | **Surname** | **Affiliation** |
| --- | --- | --- |
| Krisztina | Amrein | János Szentágothai Research Centre, University of Pécs, Pécs, Hungary. |
| Nada | Andelic | Division of Surgery and Clinical Neuroscience, Department of Physical Medicine and Rehabilitation, Oslo University Hospital and University of Oslo, Oslo, Norway. |
| Lasse | Andreassen | Department of Neurosurgery, University Hospital Northern Norway, Tromso, Norway. |
| Audny | Anke | Department of Physical Medicine and Rehabilitation, University Hospital Northern Norway, Tromso, Norway. |
| Philippe | Azouvi | Raymond Poincare hospital, Assistance Publique – Hopitaux de Paris, Paris, France. |
| Bo-Michael | Bellander | Department of Neurosurgery & Anesthesia & intensive care medicine, Karolinska University Hospital, Stockholm, Sweden. |
| Habib | Benali | Anesthesie-Réanimation, Assistance Publique – Hopitaux de Paris, Paris, France. |
| Andras | Buki | Örebro University, School of Medical Sciences, Örebro, Sweden, János Szentágothai Research Centre, University of Pécs, Pécs, Hungary. |
| Alessio | Caccioppola | Neuro ICU, Fondazione IRCCS Cà Granda Ospedale Maggiore Policlinico, Milan, Italy. |
| Emiliana | Calappi | Neuro ICU, Fondazione IRCCS Cà Granda Ospedale Maggiore Policlinico, Milan, Italy. |
| Marco | Carbonara | Neuro ICU, Fondazione IRCCS Cà Granda Ospedale Maggiore Policlinico, Milan, Italy. |
| Giuseppe | Citerio | Università Milano Bicocca; NeuroIntensive Care, ASST di Monza, Milan, Italy; Monza, Italy. |
| Hans | Clusmann | Department of Neurosurgery, Medical Faculty RWTH Aachen University, Aachen, Germany. |
| Mark | Coburn | Department of Anaesthesiology, University Hospital of Aachen and Department of Anesthesiology and Intensive Care Medicine, University Hospital Bonn, Aachen, Germany; Bonn Germany. |
| Jonathan | Coles | Department of Anesthesia & Neurointensive Care, Cambridge University Hospital NHS Foundation Trust, Cambridge, UK. |
| Marta | Correia | Radiology/MRI department, MRC Cognition and Brain Sciences Unit, Cambridge, UK. |
| Endre | Czeiter | Department of Neurosurgery, Medical School, University of Pécs, Hungary and Neurotrauma Research Group, János Szentágothai Research Centre, University of Pécs, Pécs, Hungary. |
| Véronique | De Keyser | Department of Neurosurgery, Antwerp University Hospital and University of Antwerp, Edegem, Belgium. |
| Vincent | Degos | Anesthesie-Réanimation, Assistance Publique – Hopitaux de Paris, Paris, France. |
| Bart | Depreitere | Department of Neurosurgery, University Hospitals Leuven, Leuven, Belgium. |
| Live | Eikenes | Department of Circulation and Medical Imaging, Norwegian University of Science and Technology, NTNU, Trondheim, Norway. |
| Erzsébet | Ezer | Department of Anaesthesiology and Intensive Therapy, University of Pécs, Pécs, Hungary. |
| Kelly | Foks | Department of Neurology, Erasmus MC, Rotterdam, the Netherlands. |
| Shirin | Frisvold | Department of Anesthesiology and Intensive care, University Hospital Northern Norway, Tromso, Norway. |
| Damien | Galanaud | Anesthesie-Réanimation, Assistance Publique – Hopitaux de Paris, Paris, France. |
| Alexandre | Ghuysen | Emergency Department, CHU, Liège, Belgium. |
| Ben | Glocker | Department of Computing, Imperial College London, London, UK. |
| Asta | Haberg | Department of Neuromedicine and Movement Science, Norwegian University of Science and Technology, NTNU; Department of Physical Medicine and Rehabilitation, St.Olavs Hospital, Trondheim University Hospital, Trondheim, Norway. |
| Iain | Haitsma | Department of Neurosurgery, Erasmus MC, Rotterdam, the Netherlands. |
| Eirik | Helseth | Department of Neurosurgery, Oslo University Hospital, Oslo, Norway. |
| Peter J. | Hutchinson | Division of Neurosurgery, Department of Clinical Neurosciences, Addenbrooke’s Hospital & University of Cambridge, Cambridge, UK. |
| Evgenios | Kornaropoulos | Division of Anaesthesia, University of Cambridge, Addenbrooke’s Hospital, Cambridge, UK. |
| Noémi | Kovács | Hungarian Brain Research Program - Grant No. KTIA_13_NAP-A-II/8, University of Pécs, Pécs, Hungary. |
| Ana | Kowark | Department of Anaesthesiology, University Hospital of Aachen, Aachen, Germany. |
| Steven | Laureys | Cyclotron Research Center , University of Liège, Liège, Belgium. |
| Didier | Ledoux | Cyclotron Research Center , University of Liège, Liège, Belgium. |
| Hester | Lingsma | Department of Public Health, Erasmus Medical Center-University Medical Center, Rotterdam, The Netherlands. |
| Andrew I.R. | Maas | Department of Neurosurgery, Antwerp University Hospital and University of Antwerp, Edegem, Belgium. |
| Geoffrey | Manley | Department of Neurological Surgery, University of California, San Francisco, California, USA. |
| David K. | Menon | Division of Anaesthesia, University of Cambridge, Addenbrooke’s Hospital, Cambridge, UK. |
| Tomas | Menovsky | Department of Neurosurgery, Antwerp University Hospital and University of Antwerp, Edegem, Belgium. |
| Benoit | Misset | Cyclotron Research Center, University of Liège, Liège, Belgium. |
| Visakh | Muraleedharan | Karolinska Institutet, INCF International Neuroinformatics Coordinating Facility, Stockholm, Sweden. |
| Ingeborg | Nakken | Department of Radiology and Nuclear Medicine, St.Olavs Hospital, Trondheim University Hospital, Trondheim, Norway. |
| Virginia | Newcombe | Division of Anaesthesia, University of Cambridge, Addenbrooke’s Hospital Cambridge, UK. |
| Wibeke | Nordhøy | Department of Diagnostic Physics, Clinic of Radiology and Nuclear Medicine, Oslo University Hospital, Oslo, Norway. |
| József | Nyirádi | János Szentágothai Research Centre, University of Pécs, Pécs, Hungary. |
| Fabrizio | Ortolano | Neuro ICU, Fondazione IRCCS Cà Granda Ospedale Maggiore Policlinico, Milan, Italy. |
| Paul M. | Parizel | David Hartley Chair of Radiology, Royal Perth Hospital (RPH) & University of Western Australia (UWA), Perth, WA 6000, Australia. |
| Vincent | Perlbarg | Anesthesie-Réanimation, Assistance Publique – Hopitaux de Paris, Paris, France. |
| Paolo | Persona | Department of Anesthesia & Intensive Care, Azienda Ospedaliera Università di Padova, Padova, Italy. |
| Wilco | Peul | Dept. of Neurosurgery, Leiden University Medical Center, Leiden, The Netherlands and Dept. of Neurosurgery, Medical Center Haaglanden, The Hague, The Netherlands. |
| Jussi P. | Posti | Division of Clinical Neurosciences, Department of Neurosurgery and Turku Brain Injury Centre, Turku University Hospital and University of Turku, Turku, Finland. |
| Louis | Puybasset | Department of Anesthesiology and Critical Care, Pitié -Salpêtrière Teaching Hospital, Assistance Publique, Hôpitaux de Paris and University Pierre et Marie Curie, Paris, France. |
| Sophie | Richter | Division of Anaesthesia, University of Cambridge, Addenbrooke’s Hospital Cambridge, UK. |
| Cecilie | Roe | Department of Physical Medicine and Rehabilitation, Oslo University.Hospital/University of Oslo, Oslo, Norway |
| Olav | Roise | Division of Orthopedics, Oslo University Hospital; Institute of Clinical Medicine, Faculty of medicine, University of Oslo, Oslo, Norway |
| Rolf | Rossaint | Department of Anaesthesiology, University Hospital of Aachen, Aachen, Germany |
| Sandra | Rossi | Department of Anesthesia & Intensive Care, Azienda Ospedaliera Università di Padova Padova, Italy |
| Daniel | Rueckert | Department of Computing, Imperial College London, London, UK |
| Ranjit D. | Singh | University Neurosurgical Centre Holland, Leiden University Medical Centre, Haaglanden Medical Centre, and Haga Teaching Hospital, Leiden-The Hague, Netherlands. |
| Toril | Skandsen | Department of Neuromedicine and Movement Science, Norwegian University of Science and Technology, NTNU; Department of Physical Medicine and Rehabilitation, St.Olavs Hospital, Trondheim University Hospital, Trondheim, Norway. |
| Abayomi | Sorinola | Department of Neurosurgery, University of Pécs, Pécs, Hungary. |
| Emmanuel | Stamatakis | Division of Anaesthesia, University of Cambridge, Addenbrooke’s Hospital Cambridge, UK. |
| Ewout W. | Steyerberg | Department of Public Health, Erasmus Medical Center-University Medical Center; Dept. of Department of Biomedical Data Sciences, Leiden University Medical Center, Rotterdam, The Netherlands; Leiden, The Netherlands. |
| Nino | Stocchetti | Department of Pathophysiology and Transplantation, Milan University, and Neuroscience ICU, Fondazione IRCCS Cà Granda Ospedale Maggiore Policlinico, Milano, Italy. |
| Riikka | Takala | Perioperative Services, Intensive Care Medicine and Pain Management, Turku University Hospital and University of Turku, Turku, Finland. |
| Viktória | Tamás | Department of Neurosurgery, University of Pécs, Pécs, Hungary. |
| Olli | Tenovuo | Department of Clinical Neurosciences and Turku Brain Injury Centre, Turku University Hospital and University of Turku, Turku, Finland. |
| Zoltán | Vámos | Department of Anaesthesiology and Intensive Therapy, University of Pécs, Pécs, Hungary. |
| Gregory | Van der Steen | Department of Neurosurgery, Antwerp University Hospital and University of Antwerp, Edegem, Belgium. |
| Inge A. | van Erp | University Neurosurgical Centre Holland, LUMC, HMC, HAGA, Leiden and The Hague, The Netherlands. |
| Wim | Van Hecke | icoMetrix NV, Leuven, Belgium. |
| Thijs | Vande Vyvere | icoMetrix NV, Leuven, Belgium. |
| Jan | Verheyden | icoMetrix NV, Leuven, Belgium. |
| Anne | Vik | Department of Neuromedicine and Movement Science, Norwegian University of Science and Technology, NTNU; Department of Neurosurgery, St.Olavs Hospital, Trondheim University Hospital, Trondheim, Norway. |
| Victor | Volovici | Department of Neurosurgery, Erasmus MC Rotterdam, The Netherlands. |
| Lars T. | Westlye | Norwegian Centre for Mental Disorders Research (NORMENT), Division of Mental Health and Addiction, Oslo University Hospital and Institute of Clinical Medicine, University of Oslo and Department of Psychology, University of Oslo, Oslo, Norway. |
| Daniel | Whitehouse | Division of Anaesthesia, University of Cambridge, Addenbrooke’s Hospital, Cambridge. |
| Guy | Williams | Wolfson Brain Imaging Centre, University of Cambridge. |
| Stefan | Winzeck | Department of Computing, Imperial College London, London, UK  Division of Anaesthesia, University of Cambridge, Addenbrooke’s Hospital, Cambridge. |
| Peter | Ylén | VTT Technical Research Centre, Tampere, Finland. |
| Tommaso | Zoerle | Neuro ICU, Fondazione IRCCS Cà Granda Ospedale Maggiore Policlinico, Milan, Italy. |
